# Immunosuppressive Traits of the Hybrid Epithelial/Mesenchymal Phenotype

**DOI:** 10.3389/fimmu.2021.797261

**Published:** 2021-12-15

**Authors:** Sarthak Sahoo, Sonali Priyadarshini Nayak, Kishore Hari, Prithu Purkait, Susmita Mandal, Akash Kishore, Herbert Levine, Mohit Kumar Jolly

**Affiliations:** ^1^ Undergraduate Program, Indian Institute of Science, Bangalore, India; ^2^ Centre for BioSystems Science and Engineering, Indian Institute of Science, Bangalore, India; ^3^ College for Integrated Studies, University of Hyderabad, Hyderabad, India; ^4^ Department of Computer Science & Engineering, Sri Sivasubramaniya Nadar (SSN) College of Engineering, Chennai, India; ^5^ Center for Theoretical Biological Physics, Northeastern University, Boston, MA, United States; ^6^ Departments of Physics and Bioengineering, Northeastern University, Boston, MA, United States

**Keywords:** hybrid epithelial/mesenchymal, PD-L1, immune evasion, multistability, epithelial- mesenchymal transition (EMT)

## Abstract

Recent preclinical and clinical data suggests enhanced metastatic fitness of hybrid epithelial/mesenchymal (E/M) phenotypes, but mechanistic details regarding their survival strategies during metastasis remain unclear. Here, we investigate immune-evasive strategies of hybrid E/M states. We construct and simulate the dynamics of a minimalistic regulatory network encompassing the known associations among regulators of EMT (epithelial-mesenchymal transition) and PD-L1, an established immune-suppressor. Our simulations for the network consisting of SLUG, ZEB1, miR-200, CDH1 and PD-L1, integrated with single-cell and bulk RNA-seq data analysis, elucidate that hybrid E/M cells can have high levels of PD-L1, similar to those seen in cells with a full EMT phenotype, thus obviating the need for cancer cells to undergo a full EMT to be immune-evasive. Specifically, in breast cancer, we show the co-existence of hybrid E/M phenotypes, enhanced resistance to anti-estrogen therapy and increased PD-L1 levels. Our results underscore how the emergent dynamics of interconnected regulatory networks can coordinate different axes of cellular fitness during metastasis.

## Introduction

The progression of cancer relies on a complex interplay of various cell autonomous and non-cell autonomous phenomena. The latter includes the well-established fact that cancer cells can proactively create a microenvironment that aids their own survival. One of the employed strategies is to suppress various arms of immune system that can lead to cancer cell elimination (1). For instance, some tumor cells can inhibit the functions of effector T (T_eff_) cells and/or induce a population of tolerogenic cells that ultimately result in the immune escape of the tumor. They can also facilitate accumulation of immune suppressive cells such as regulatory T (T_reg_) cells, myeloid derived suppressor cells (MDSCs) and M2 macrophages/tumor-associated macrophages (TAMs), leading to enhanced tumor growth (1). Understanding these strategies of tumor-driven reprogramming of the microenvironment would be a major step towards more effective guiding of various therapeutic interventions.

In addition to reprogramming the immune cells in the stroma, tumors employ cell autonomous mechanisms that help them directly evade cytotoxic CD8 T cells. A key mechanism *via* which tumor cells achieve this evasion is *via* the expression of programmed death-ligand 1 transmembrane protein (PD-L1) on their cell membranes (2). The binding of PD-L1 to PD-1 receptors on activated T cells drives the exhaustion of these T cells, reducing their cytotoxic abilities (3). In cancer cells, a multitude of molecular players modulate PD-L1 levels at various regulatory stages (2). Of interest here is the finding that PD-L1 levels can be increased as cells go through an Epithelial-Mesenchymal Transition (EMT) and consequently gain the ability to migrate and invade (4–13), as noted in *in vitro* models as well as in patient samples across cancer types. It is, however, important to note here that tumours from metastatic sites have often been shown to have relatively low levels of PD-L1 (14, 15). The mechanistic reason for such an observation is still unknown.

The process of EMT, however, is not typically a binary switch, as had been tacitly assumed in these earlier works. Instead, cells can stably maintain one or more hybrid epithelial/mesenchymal (E/M) phenotypes that can be much more metastatic than cells in a ‘full EMT’ or ‘extremely mesenchymal’ state (16). Besides, hybrid E/M phenotypes across cancers can be resilient to various therapies (16). However, the immune evasive properties of hybrid E/M states are relatively poorly understood.

In this study, we identify a core regulatory network that helps us elucidate the immune evasive properties of different phenotypes along the epithelial-hybrid-mesenchymal spectrum. We simulate the proposed gene regulatory network, consisting of EMT players (ZEB1, SLUG, miR-200 and E-cadherin) and their coupling with PD-L1, using RACIPE (17) so as to elucidate the network’s steady state characteristics. Our simulations indicate that hybrid E/M phenotypes are extremely likely to exhibit high PD-L1 levels, similar to those seen in mesenchymal cells, thus obviating the need to undergo a full EMT to develop immunosuppression. Moreover, the switch from an epithelial/low-PDL1 state to hybrid/high-PDL1 or mesenchymal/high-PDL1 state is reversible, i.e., while EMT can induce PD-L1 levels, MET can reduce them. Specifically, for ER+ breast cancer, we expand our network to include elements of estrogen receptor signaling implicated in tamoxifen resistance. This allows us to show how the acquisition of phenotypic resistance to targeted therapy such as tamoxifen can co-occur with high PD-L1 levels, thus enabling cross-resistance and enhancing cancer cell fitness during metastasis. Our model predictions are validated by extensive analysis of transcriptomic datasets across multiple cancers at both bulk and single cell levels.

## RESULTS

### Hybrid E/M and Mesenchymal Cell States Are More Likely to Exhibit High PD-L1 Levels

**Figure 1:**
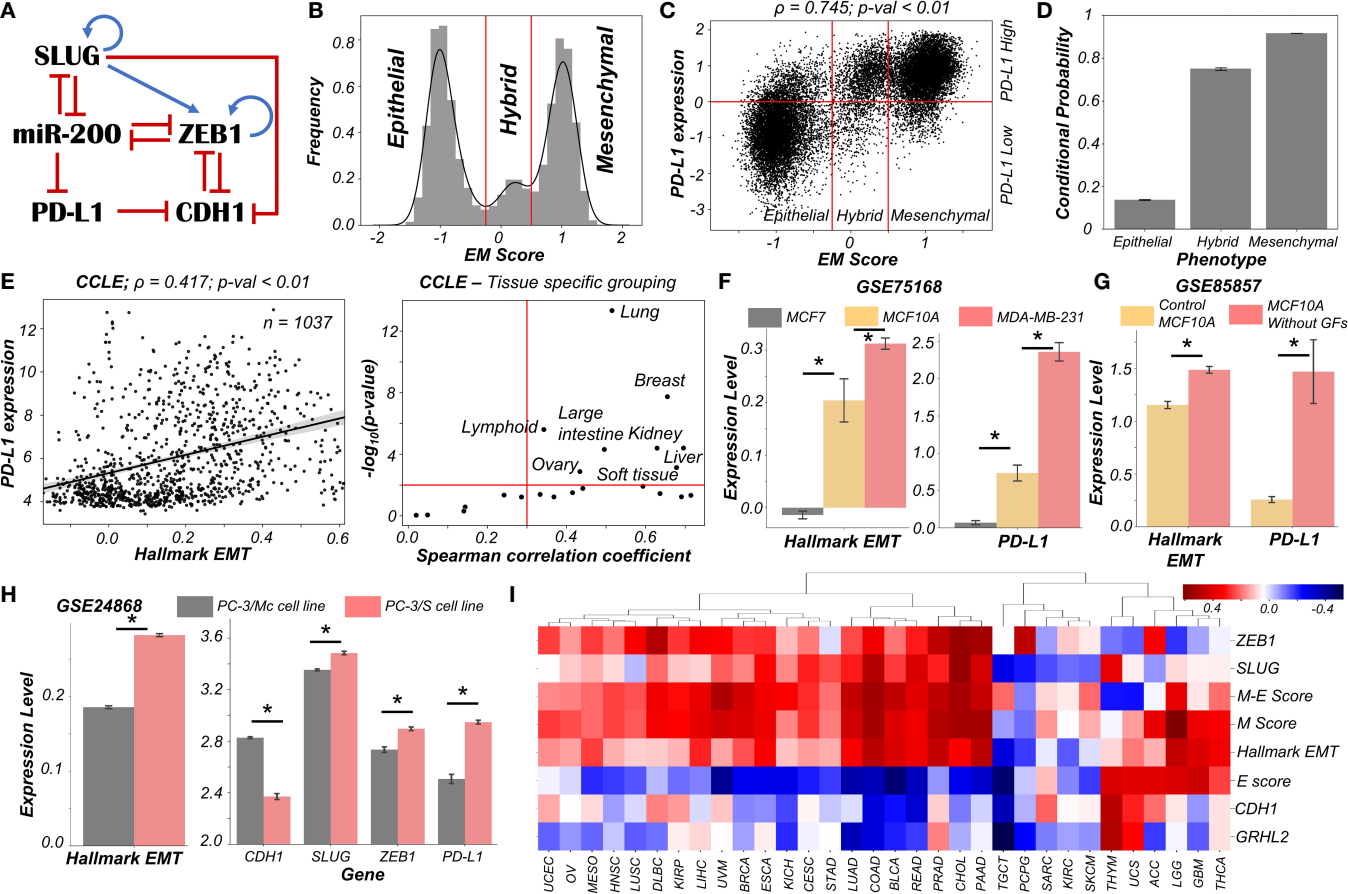
Dynamics of regulatory network coupling EMT with PD-L1. **(A)** Regulatory network (GRN) capturing the interplay of EMT regulators coupled with PD-L1. Blue arrows stand for activation links, red hammers for inhibitory links. **(B)** Density histogram of EM Score fitted with kernel density estimate showing a trimodal distribution. Red lines show the partition between phenotypes: Epithelial, Hybrid, and Mesenchymal. **(C)** Scatter plot of PD-L1 expression and EM score. Horizontal red line shows the partition between PDL1 expression level being high vs. low. Vertical red lines show the partition between phenotypes: Epithelial, Hybrid, and Mesenchymal based on EM score. Spearman’s correlation coefficient (ρ) and corresponding p-value (p-val) have been reported. **(D)** Bar plot representing the conditional probability of a phenotype being PD-L1 high given that it belongs to a given EMT phenotype. Error bars denote standard deviation calculated on three independent simulations. **(E)** Scatter plot showing correlation between PD-L1 levels and the Hallmark EMT signature in cell lines from CCLE. Spearman’s correlation coefficient (ρ) and corresponding p-value (p-val) are reported (left panel). Splitting CCLE cell lines reveals tissues that show a strong significant correlation (ρ > 0.3 and p-val < 0.01) (right panel). **(F)** Activity levels of Hallmark EMT and PD-L1 expression levels in 3 breast cancer cell lines (GSE75168). **(G)** Activity/Expression levels of Hallmark EMT and PD-L1 levels in MCF10A breast cancer cells treated with or without growth factors (GSE85857). **(H)** Activity/Expression levels of Hallmark EMT and PD-L1 levels in two prostate cancer sub-lines of PC3 with different EMT status (GSE24868). **(I)** Heatmap showing the Spearman’s correlation coefficients between the different EM metrics and EMT associated genes and PD-L1 levels across 32 different cancer types in TCGA. * denotes a statistically significant difference (p-val < 0.05) between the represented groups assessed by a two-tailed Students t-test assuming unequal variances.

Capturing the essence of biological processes *via* mechanism-based mathematical modelling can be a daunting task, given the vast complexity of biological systems. Identifying an appropriately sized gene regulatory network that incorporates the essential features of the underlying biological mechanism at hand in a minimalistic, yet informative manner is a key first step. To that extent, we started out with a core set of four well-reported biomolecules and their interactions that can capture the non-binary nature of the epithelial-mesenchymal plasticity (EMP) spectrum and the ability of different phenotypes to modulate PD-L1 levels: ZEB1, miR-200, CDH1 (E-cadherin) and SLUG (**Figure 1A**). Mutual inhibition between ZEB1 and miR-200, together with ZEB1 self-activation can enable epithelial, mesenchymal and hybrid epithelial/mesenchymal (E/M) phenotypes (18). SLUG has been reported to specifically associate with hybrid E/M phenotype both in experimental and computational analysis (19, 20).

High levels of ZEB1 and SLUG with low levels of CDH1 and miR-200 are frequently attributed to a mesenchymal phenotype, while high levels of CDH1 and miR-200 with concurrent reduced levels of ZEB1 and SLUG usually associates with an epithelial phenotype (21). Interactions between ZEB1, miR-200, CDH1, and SLUG have been extensively studied (20–23). Furthermore, miR-200 has been known to directly inhibit PD-L1 by binding to 3’ untranslated region of its mRNA (4). PD-L1 can, in turn, repress the levels of CDH1 *via* indirect mechanisms (24–27) (**Figure 1A**).

We used RACIPE (17) to generate *in-silico* steady state gene expression values enabled by this gene regulatory network (**Methods**). RACIPE simulates a given gene regulatory network as a set of coupled ordinary differential equations (ODEs), with parameters sampled from biologically relevant ranges. The ensemble of resultant steady states is indicative of the possible phenotypes allowed by the network topology. To quantify the cellular phenotype of given steady state solution, we defined an EM score from z-normalized expression values of ZEB1, SLUG, miR-200, and CDH1. The higher the EM score, the more mesenchymal is the corresponding phenotype. A histogram of these scores showed a clear trimodal distribution, which can be construed as consisting of epithelial, hybrid E/M, and mesenchymal phenotypes; these assignments can be confirmed by PCA plots (**Figures 1B**, **S1A, B**). Subsequently, we also observed a bimodal distribution of PD-L1 levels (**Figure S1C**) where high levels of PD-L1 can be viewed as an immune-evasive state while low PD-L1 denotes an immune-sensitive tumor cell state (28).

Next, we investigated the association between the EM scores and PD-L1 levels and observed a strong positive correlation between them (ρ = 0.745; p-value < 0.01) (**Figure 1C**). Conditional probability analysis shows that only a small percentage (~15%) of epithelial cells were PD-L1+. In contrast, a much larger percentage of hybrid E/M (~70%) and mesenchymal (~90%) cells were PD-L1+ (**Figures 1D**, **S1D**). Consistently, PD-L1 was found to negatively correlate with CDH1 but positively with ZEB1 and SLUG (**Figure S1E**). Further, ZEB1, SLUG, and PD-L1 all had intermediate levels in hybrid E/M states compared to extreme states – epithelial and mesenchymal (**Figure S1F**). Together, these results suggest that cells undergoing either a partial or full EMT can upregulate their levels of PD-L1 and consequently can exhibit immune evasion.

To validate these model predictions, we analyzed pan-cancer gene expression datasets such as CCLE (Cancer Cell Line Encyclopaedia), where we observed the ssGSEA scores of EMT to be positively correlated with PD-L1 levels (**Figure 1E**; left). A tissue-specific analysis revealed a majority (16 out of 22) of cancers exhibited a strong correlation (ρ > 0.3) between EMT and PD-L1 expression (**Figure 1E**; right). Next, we investigated more specific scenarios. For instance, three breast cancer cell lines along the EMP spectrum – MCF7 (epithelial), MCF10A (hybrid E/M) and MDA-MB-231 (mesenchymal) (29, 30) – showed consistent trends with PD-L1 levels with MCF7 < MCF10A < MDA-MB-231 (**Figure 1F**). This pattern was recapitulated in an analysis of TCGA luminal breast cancer samples (**Figure S1G**). Furthermore, MCF10A cells, when driven to a more mesenchymal phenotype upon growth factor depletion (31), showed a concomitant increase in the levels of PD-L1 (**Figure 1G**). Similarly, comparing two sub-lines of prostate cancer cells PC-3 (32), the more mesenchymal one (PC-3/S) showed higher levels of PD-L1, ZEB1, and SLUG relative to the hybrid E/M PC-3/Mc cells (**Figure 1H**). A positive correlation between EMT signature and PD-L1 levels was also seen in A549 lung adenocarcinoma cells induced to undergo EMT (**Figure S1H**), suggesting a pan-cancer association of EMT with PD-L1 levels.

Finally, we analyzed TCGA patient cohort datasets for all the above-mentioned features. We calculated the Spearman’s correlation coefficient between PD-L1 expression levels with those of CDH1, GRHL2, SLUG, and ZEB1 as well as with a Hallmark EMT signature, and epithelial and mesenchymal specific signatures (**Figures 1I**, **S2**). A majority of cancers (21 out of 32) showed a strong positive correlation of PD-L1 with the mesenchymal related metrics (ZEB1, SLUG, Hallmark EMT, mesenchymal score, and M-E score) while showing an intermediate to strong negative correlation with the epithelial ones (CDH1, GRHL2, and epithelial score), thereby endorsing our model predictions. Intriguingly, 6 cancer types (THYM, UCS, ACC, LGG, GBM, and THCA) showed a positive correlation of PD-L1 with both epithelial and mesenchymal signatures, highlighting a possible association of highest PD-L1 levels with the hybrid E/M phenotype. It should be noted that our model does not completely preclude the association of an epithelial state with high PD-L1 levels, although the likelihood of such association is relatively low (**Figure 1C**). This infrequent association may underlie context-specific behavior of epithelial tumours (such as Thymic epithelial tumours) that also can show high PD-L1 positivity (**Figure 1I**) (33).

Overall, *in silico* predictions, supported by analysis of *in vitro* and patient data, suggests that a change in the EMP status of the cell is positively associated with PD-L1 levels across various cancer types. These results clearly indicate the likelihood of the hybrid E/M phenotype being (almost) as immune evasive as the mesenchymal phenotype.

### Traversal of Cells on the EMP Spectrum Can Alter the PD-L1 Status of the Cells

**Figure 2:**
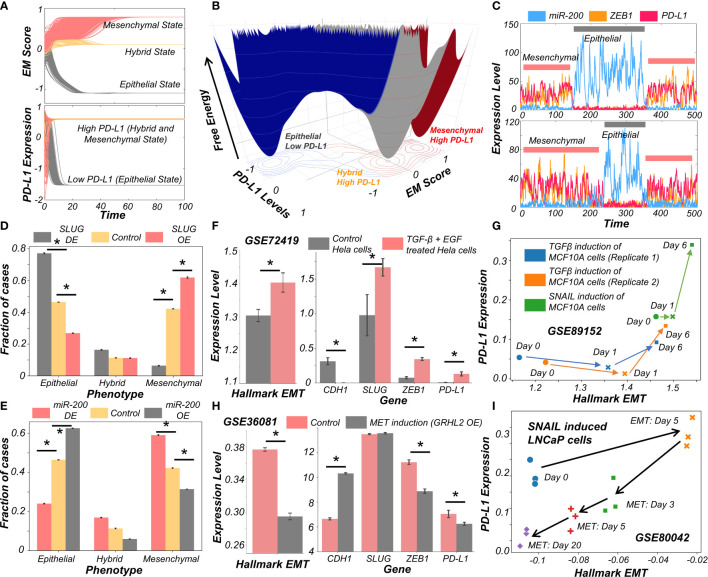
Evidence for causal links between EMT associated genes and PD-L1 levels. **(A)** Dynamics of EM score and PD-L1 showing presence of epithelial, hybrid, and mesenchymal phenotypes and their corresponding PD-L1 levels, when simulated from multiple initial conditions. **(B)** Probability landscape on the PD-L1 and EM score plane, with the valleys representing the stable states possible in the system. Three distinct states – Epithelial/PD-L1 low, Hybrid E-M/PD-L1 high, and Mesenchymal/PD-L1 high – are observed. **(C)** Stochastic simulations of gene regulatory network *via* sRACIPE showing spontaneous switching between different states. **(D)** Simulation results showing the fraction of cases of epithelial, hybrid, and mesenchymal phenotypes under control (yellow), SLUG DE (grey) and SLUG OE (orange) conditions. **(E)** Same as D but for miR-200 OE (grey) and miR-200 OE (orange) conditions. **(F)** Activity/Expression levels of Hallmark EMT and PD-L1 levels in Hela cells induced to undergo EMT (GSE72419). **(G)** Two-dimensional Hallmark EMT and PD-L1 plot showing trajectory of MCF10A cells induced with TGFβ or SNAIL (GSE89152). **(H)** Activity/Expression levels of Hallmark EMT and PD-L1 levels in HMLE cells where MET has been induced via overexpression of GRHL2 (GSE36081). **(I)** Two-dimensional Hallmark EMT and PD-L1 plot showing trajectory of LNCaP prostate cancer cells that have been induced with SNAIL to undergo EMT followed by removal of signal to induce MET (GSE80042). * denotes p-val < 0.05 as assessed by a two-tailed Students t-test assuming unequal variances.

After establishing a pan-cancer correlation between more mesenchymal status and higher PD-L1 levels, we examined a causal connection between them. We simulated the set of coupled ODEs for a representative model whose parameter set gave rise to tristability. We started with diverse initial conditions and observed convergence to three distinct EM states (**Figure 2A**). Corresponding PD-L1 levels followed the previously observed patterns with epithelial state showing the least PD-L1 levels, while both the hybrid E/M and mesenchymal showing nearly equal levels of PD-L1 higher than those of the epithelial state (**Figure 2A**). Stochastic simulations for this parameter set created a landscape indicative of the steady states under the influence of biological noise. This landscape revealed the co-existence of three states (shown by valleys) – (Epithelial, PD-L1 low), (Hybrid E/M, PD-L1 high) and (Mesenchymal, PD-L1 high) (**Figure 2B**), depicting that as cells change their EM status, their corresponding PD-L1 levels are also altered. In another parameter set to study the stochastic dynamics of this network (34), we observed spontaneous switches between epithelial and mesenchymal states with a concurrent change in the levels of PD-L1 (**Figure 2C**). Together, this analysis points towards the possibility of a switch like behavior in acquisition of an immune evasive phenotype as the cells undergo EMT.

To characterize the impact of perturbations on our core regulatory network we simulated the scenarios of EMT induction and MET induction. EMT was induced by down expression (DE) of miR-200 or over expression (OE) of SLUG; conversely, MET was induced by OE of miR-200 or DE of SLUG (23). SLUG-OE or miR-200 DE increased the proportion of mesenchymal cell states with a concurrent decrease in epithelial cases (**Figures 2D, E**, **S3A–E**). This change resulted in a significant increase in PD-L1 levels (**Figure S3C, F**). Opposite trends were observed in the cases of MET induction *via* miR-200 or SLUG-DE, with resultant changes in PD-L1 levels (**Figures 2D, E**).

Next, we investigated whether our model prediction about a concurrent switch in EM status and PD-L1 levels is supported by experiment, through analyzing corresponding transcriptomic data. HeLa cells treated with TGFβ and EGF were thereby induced to undergo EMT, evident by increases in SLUG and ZEB1 levels, as well in the activity (as measured *via* ssGSEA; see *Material and Methods*) of the Hallmark EMT gene set (**Figure 2F**). In treated cells, CDH1 levels were significantly decreased while PD-L1 levels were increased (**Figure 2F**). This phenomenon of EMT-driven increase in PD-L1 was also seen in non-cancerous cells where TGFβ treatment of primary airway epithelial cells led to upregulation of EMT and PD-L1 (**Figure S3G**), indicating that this association between EMT and PD-L1 levels need not be restricted to cancer cells. Furthermore, we compared the profiles of triple negative breast cancer cells DKAT when grown in a medium supporting epithelial growth (MEGM) vs when grown in a medium containing stromal factors (SCGM). These have been shown to differ in their EM status: while culturing in SCGM facilitated a mesenchymal phenotype, that in MEGM drove an epithelial one (GSE33146). Consistently, SLUG, ZEB1 and PD-L1 levels were significantly higher in cells grown in SCGM rather than in MEGM (**Figure S3H**). Furthermore, in a time course experiment where EMT was induced in A549 lung cancer cells by treatment with TGFβ over 96 hours, the failure of sustained expression of ZEB1 was correlated with a visibly lower level of PD-L1 levels, hinting towards a likely causal role of ZEB1 in enhanced PD-L1 expression levels. (**Figure S3I**). Next, we analyzed a set of experiments in which MCF10A cells were induced to undergo EMT either *via* TGFβ application or by induced overexpression of SNAIL. This time course experiment resulted in an increase of activity of Hallmark EMT genes and PD-L1, irrespective of cells’ initial EM status (**Figure 2G**).

Finally, we asked whether induction of MET can decrease the levels of PD-L1 in cancer cells. HMLE cells, upon overexpression of MET-inducing factor GRHL2, displayed a more epithelial state (increased CDH1, decreased ZEB1 and decreased hallmark EMT signature) with a substantial drop seen in PD-L1 levels (**Figure 2H**), indicating that EMT-driven changes in PD-L1 levels are reversible. Similar observations were made for PD-L1 levels in LNCaP prostate cancer cells which were first induced to undergo EMT and subsequently were induced to undergo MET. A two-dimensional plot of EM score and PD-L1 levels revealed an increase in PD-L1 as EMT was induced, and a subsequent decrease when MET was induced **(** **Figure 2I** **)**. Intriguingly, the cell population did not retrace its original path during MET induction, indicative of hysteresis in the system (35). The overall levels of PD-L1 were lower at the end of 20 days of MET than for the uninduced cells themselves, suggesting that MET induction can reset the baseline PD-L1 levels upon a cycle of EMT and MET. The fact that the extent of MET and its consequent effect on PD-L1 levels are hysteretic in nature (i.e. cells do not return to their pre-EMT starting point) can possibly explain why tumours at metastatic sites which have undergone a cycle of EMT and MET can show lower levels of PD-L1 as compared to primary tumours (14, 15). Collectively, these results underscore that induction of EMT or MET in cancer cells (and possibly other cells as well) can regulate their immune evasion status through altered levels of PD-L1.

### Various Signalling Pathways Can Either Independently or in Concert Modulate the Immune Evasive Properties of Cancer Cells on the EMP Spectrum

**Figure 3:**
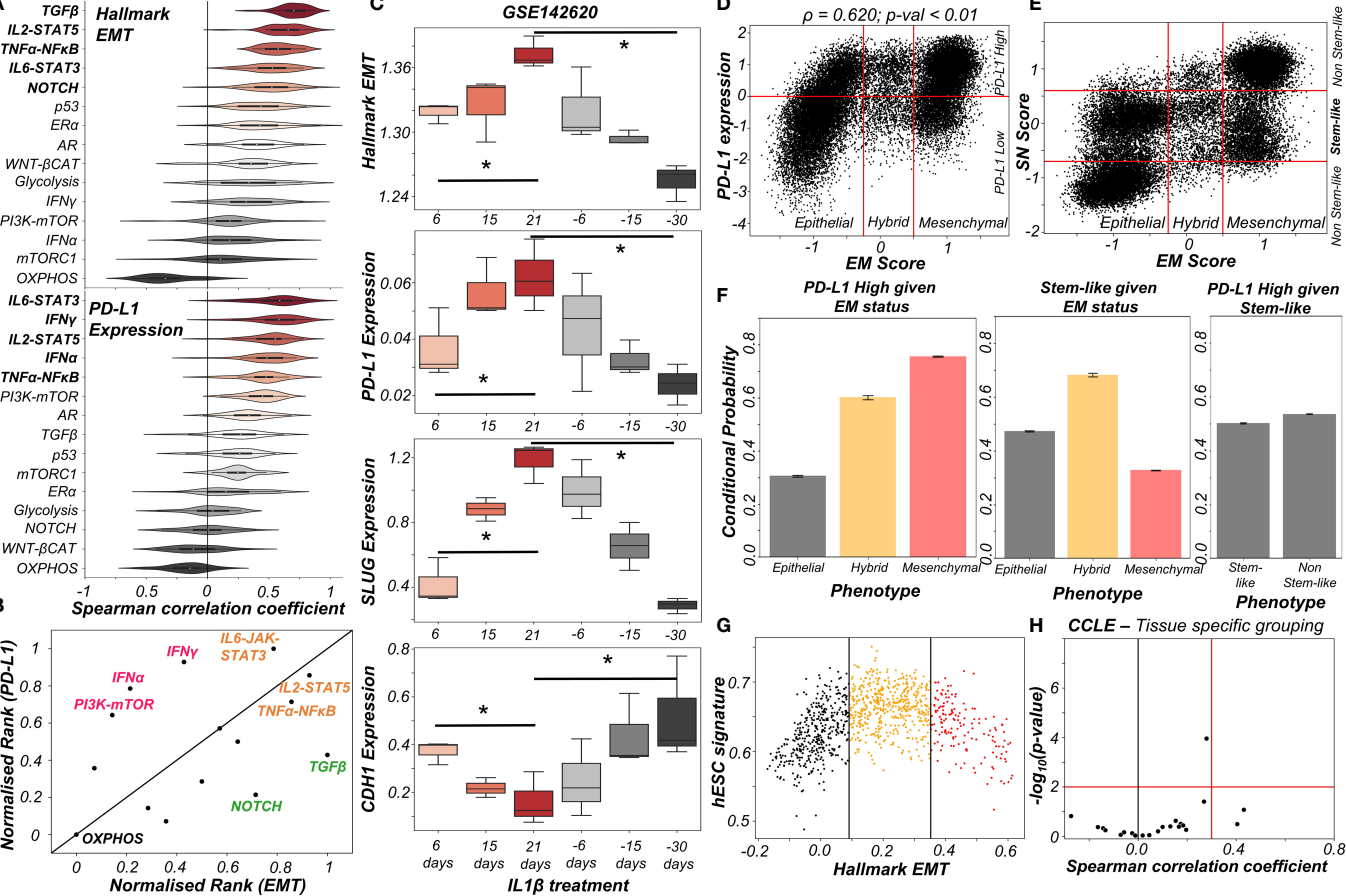
Signalling pathways and biological processes that can affect PD-L1 and/or EMT. **(A)** Violin plots of Spearman’s correlation values of different signalling pathways with Hallmark EMT programme (top) or with PD-L1 levels (bottom) ordered by corresponding median values across 27 cancer types in TCGA. **(B)** Scattered plot between normalized ranks of signalling pathways with the EMT programme and with PD-L1 expression levels. Signalling pathways hypothesized to be specific to EMT programme are labelled in green, those specific for PD-L1 highlighted in pink and those with both in orange. **(C)** Activity/expression levels of Hallmark EMT, PD-L1, SLUG, and CDH1 levels in lung cancer cells treated with IL-1β and subsequent removal of signal (GSE142620). **(D)** Scatter plot of PD-L1 expression and EM score. Horizontal red line shows the partition between PD-L1 expression level being high vs low for the circuit in **Figure S4C**. Vertical red lines show the partition between phenotypes: Epithelial, Hybrid, and Mesenchymal based on EM score. Spearman’s correlation coefficient (ρ) and corresponding p-value (p-val) are reported. **(E)** Scatter plot of SN score and EM score showing the presence of clusters having predominantly stem-like hybrid and the presence of both stem like and non-stem like epithelial and mesenchymal cells scattered in the plane. Horizontal red lines show the partition between stem-like and non-stem-like based on SN score and EM phenotypes. **(F)** Bar plot representing conditional probability of PD-L1 being high given EM status, stem-like phenotype given EM status, and PD-L1 high given stemness status respectively. **(G)** Scatter plot showing the non-monotonic association between the hESC signature and the Hallmark EMT signature in CCLE dataset. The boundaries are determined by trisection of the entire range of Hallmark EMT signature values. **(H)** No tissue in CCLE shows a strong significant Spearman’s correlation (ρ > 0.3 and p-val < 0.01) between hESC signature and PD-L1 levels. * denotes a statistically significant difference (p-val < 0.05) between the represented groups assessed by a two-tailed Students t-test assuming unequal variances.

The above-mentioned interconnections among the EMT regulators and PD-L1 levels seldom work in isolation. Multiple signalling pathways can independently or in concert affect the EM status of cells and/or their PD-L1 expression. To investigate such effects, we calculated the degree of correlation of 15 well-defined signalling pathways with EMT and with PD-L1 levels across different cancers in the TCGA cohort (**Figure S4A**). A scatter plot of corresponding correlation coefficients revealed pan-cancer consistency in signalling pathways associated with EMT and with PD-L1 levels (*ρ* = 0.37; p-value < 0.01) (**Figure S4B**). Next, we ranked which pathways correlate most strongly with EMT signature or PD-L1 levels (**Figure 3A**). TGFβ, IL2-STAT5, TNFα-NFκB, IL6-STAT3 and NOTCH signalling were found to correlate strongly with EMT, consistent with their expected roles (36). Similarly, PD-L1 levels are most correlated with IL6-STAT3, IFNγ, IL2-STAT5, IFNα and TNFα-NFκB, all of which have been previously implicated (37). Plotting these pathways through their normalized ranks allows identifying the pan-cancer independent regulators of PD-L1 levels and EMT; for instance, TGFβ and NOTCH can be considered as more EMT-specific, while IFNγ and IFNα are PD-L1 specific modulators. IL6-JAK-STAT3, IL2-STAT5 and TNFα-NFκB pathways correlated both with PD-L1 and EMT (**Figure 3B**). IL1β is known to act partially by the NFκB pathway (38). Thus, it is not surprising to see that treatment of cancer cells with IL1β caused a concerted increase in EMT as well as in PD-L1 levels; the consistency in these trends was also visible upon withdrawal of the signal (**Figure 3C**).

The interplay between stemness and EMT has been extensively investigated (39, 40). Thus, we asked whether EMT, stemness and PD-L1 levels all vary together. To investigate this crosstalk, we simulated an extended regulatory network including stemness regulators (OCT4, miR-145, LIN28, let-7) *via* RACIPE (**Figure S4C**). A stemness window was defined based on the distribution of stemness (SN) score (**Figure S4D**). This network showed conserved trends between EM score and PD-L1 expression level and found that most hybrid E/M solutions lay within the stemness window (**Figures 3D, E**). Quantifying these trends among EMT status, stemness status and PD-L1 levels revealed that while hybrid E/M cells were very likely to exhibit both PD-L1 and enhanced stemness; the stemness status by itself (irrespective of EMT status) could not predict any association with PD-L1 (**Figure 3F**). The non-monotonic nature of association between EMT states and stemness was confirmed by pan-cancer data analysis of CCLE cell lines, where the stemness signature was most enriched in cells with hybrid E/M status (**Figures 3G**, **S4E**) while no such trend was seen for a direct association of stemness with PD-L1 levels (**Figure 3H**). Together, we conclude that while hybrid E/M cells are more stem-like and immune-evasive, these two features are likely acquired independent of one another.

### Reversible Resistance to Anti-Estrogen Therapy Can Co-Occur With an Immune Evasive Phenotype in Breast Cancer

We now look more closely at the specific case of breast cancer and the connection between drug resistance and immune evasion. The emergence of reversible resistance to targeted therapy such as tamoxifen in ER+ positive breast cancer can be modulated in part by EMT-associated players such as ZEB1, miR-200 and SLUG, by virtue of their crosstalk with the estrogen receptor alpha (ERα). A cell-state switch to a hybrid E/M or a mesenchymal phenotype can enable the acquisition of a tamoxifen resistant phenotype where ERα levels are relatively low (41, 42). Further, ERα has been reported to directly repress PD-L1 transcriptionally (43, 44), thus raising the possibility of emergence of an immune evasion phenotype due to increased PD-L1 levels in cells that are treated with anti-estrogen therapy.

**Figure 4:**
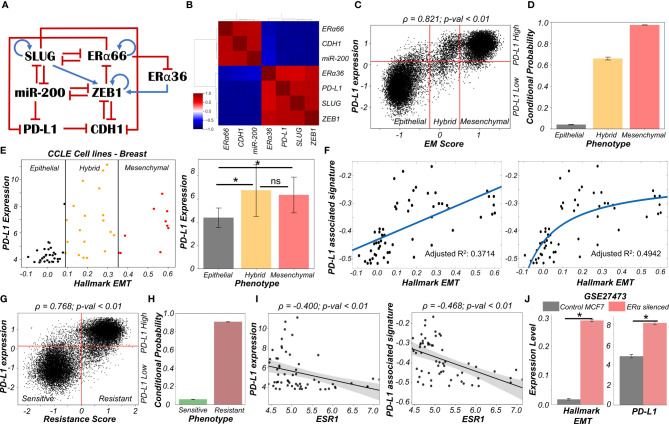
Association of high PD-L1 levels with acquisition of a reversible drug resistant phenotype in ER+ Breast cancer. **(A)** Regulatory network interplay of EMT regulators, estrogen receptor isoforms (ERα66, ERα36) coupled with PD-L1. Blue arrows stand for activation links, red hammers for inhibitory links. **(B)** Pairwise correlation matrix using Spearman correlations showing the existence of 2 “teams” of players – SLUG, ZEB1, ERα36, PD-L1 and CDH1, miR-200 and ERα66 – with mutually antagonistic associations. **(C)** Scatter plot of PD-L1 levels and EM score. Spearman’s correlation coefficient (ρ) and corresponding p-value (p-val) are reported. **(D)** Bar plot representing conditional probability of a phenotype being PD-L1 high given that it belongs to a given EMT phenotype. **(E)** Scatter plot showing correlation between PD-L1 levels and the Hallmark EMT signature in breast cancer specific cell lines from CCLE. The boundaries between epithelial, hybrid and mesenchymal phenotypes are based on trisection of the entire range of Hallmark EMT scores of all cell lines in CCLE (left). Quantification of PD-L1 levels of breast cancer cell lines belonging to different EM status (right). * denotes p-val < 0.05 as assessed by a two-tailed Students t-test assuming unequal variances. **(F)** Scatter plot showing linear vs Michaelis-Menten curve fit to a scatter plot of PD-L1 associated and Hallmark EMT signatures. **(G)** Scatter plot of PD-L1 levels and Resistance score classified as high (>0) vs low (<0). Spearman’s correlation coefficient (ρ) and corresponding p-value (p-val) are reported. **(H)** Bar plot representing conditional probability of a phenotype being PD-L1 high given that it is sensitive vs. resistant state. **(I)** Scatter plot showing a significant negative correlation between PD-L1 levels and PD-L1 associated signature and ESR1 expression levels in breast cancer cell lines from CCLE. **(J)** Activity/Expression levels of Hallmark EMT and PD-L1 levels in MCF7 ER+ breast cancer cells with control and ERα silenced cases (GSE27473). * denotes a statistically significant difference (p-val < 0.05) between the represented groups assessed by a two-tailed Students t-test assuming unequal variances. ns represents results that are not statistically significant (p-val > = 0.05).

To study the emergent properties of this system, we added following components to the regulatory network in **Figure 1A** – ERα66, the major target of anti-estrogen therapy and ERα36, a mediator of anti-estrogen therapy resistance (**Figure 4A**). The interconnections between ERα66 and ERα36 were taken from our previous study (41). Also, we included an inhibitory link from ERα66 to PD-L1 (43). We simulated this updated regulatory network in RACIPE and obtained pairwise correlations of simulated expression levels for all nodes in the network, which revealed two distinct and mutually antagonistic “teams” (45). This presumably arises due to the fact that interactions of the core EMT circuit with the estrogen response module do not exhibit any frustration (46). One team comprised EMT-inducers ZEB1 and SLUG, ERα36 and PD-L1 which were all highly correlated with one another and negatively with members of the other team which consisted of CDH1, miR-200 and ERα66 (**Figure 4B**). This pattern was consistently seen in all observed steady state solutions plotted as a heatmap, i.e., two predominant cell-states – one in which ZEB1, SLUG, ERα36, PD-L1 are high and CDH1, miR-200, ERα66 are low and *vice versa*, with a small proportion of mixed states (**Figure S5A**).

To quantify the resultant phenotypes, we computed the EM score as before, and a (tamoxifen) Resistance score (= ERα36 – ERα66) (41). The resultant distribution of EM Score was visibly trimodal while that of Resistance score and PD-L1 levels were bimodal (**Figure S5B**). Plotting the EM score and Resistance scores together identified 6 phenotypes – ES (Epithelial-Sensitive), ER (Epithelial-Resistant), HS (Hybrid-Sensitive), HR (Hybrid-Resistant), MS (Mesenchymal-Sensitive), and MR (Mesenchymal-Resistant), with ES and MR being most predominant and showed a strong positive correlation between these scores (**Figure S5C**). The association between EM score and PD-L1 levels were also similarly distributed and positively correlated (**Figure 4C**) as seen earlier (**Figure 1C**). Further, a hybrid E/M or mesenchymal phenotype was more likely to be PD-L1+ as well as tamoxifen-resistant as compared to an epithelial one (**Figures 4D**, **S5D**), consistent with previously observed trends (**Figure 1D**). These results indicate the association of properties of individual/pairs of nodes in a network remains strongly conserved, even with the addition of an extra (i.e., tamoxifen resistance) module.

Next, to validate these predictions from our simulations, we analyzed breast cancer cell lines in the CCLE dataset. We designated the cell lines as epithelial, hybrid or mesenchymal by trisecting the overall range of activity of the Hallmark EMT gene set (47) (**Figure 4E** – left panel). In accordance with our model predictions, we observed PD-L1 levels in hybrid cell lines to be significantly higher than those in epithelial ones, but comparable to those in mesenchymal ones (**Figure 4E** – right panel). A similar trend was seen for enrichment of PD-L1 associated gene set (see *Material and Methods*) (**Figure S5E**). This observation indicated the possibility that hybrid E/M breast cancer cell lines can be as immune evasive as mesenchymal cell lines. In other words, change in levels of PD-L1 is likely to occur in the earlier stages of EMT as compared to later stages. To further strengthen this hypothesis, we fitted a straight line (ax + b) and a Michaelis-Menten kind of a curve (ax/(b+x) + c) to a scatterplot between the ssGSEA scores of Hallmark EMT gene set and PD-L1 associated gene set (**Figure 4F**). A much better fit was observed in the latter case (adjusted R^2^ = 0.49) vs than a simple straight line fit (adjusted R^2^ = 0.37) (**Figure 4F**), indicating towards the possibility of a non-linear and saturating model of association between changes of cell phenotype along the EMT axis and consequent PD-L1 levels.

Having validated our simulation-based observations in breast cancer cell lines for EMT/PD-L1 association, we next investigated the axes of reversible drug resistance to anti-estrogen therapy and PD-L1 expression. Our simulations suggested a strong positive correlation between PD-L1 levels and resistance score (**Figure 4G**), such that resistant cells are largely PD-L1+ while sensitive ones are largely PD-L1- (**Figure 4H**). This prediction was validated in CCLE group of breast cancer cell lines, where both the expression levels of PD-L1 and corresponding ssGSEA scores of PD-L1 associated gene set were significantly negatively correlated to *ESR1* expression levels (**Figure 4I**). To gain a stronger causal evidence in support of our simulation results, we looked into a specific experimental dataset in which ERα was silenced in an ER+ breast cancer cell line, namely MCF7. We found that as ERα silencing led to a concurrent increase in the activity of the Hallmark EMT gene set as well as in PD-L1 levels (**Figure 4J**). This trend indicates that as the levels of ERα and consequently the ERα signalling activity falls, a coordinated increase in the mesenchymal nature of cells and PD-L1 levels ensues, thus leading to the resultant phenotype being more immune evasive.

Finally, we examined whether the association between PD-L1 levels, EMT and ERα levels seen *in vitro* and *in silico* holds true in patients with different subtypes of breast cancer. Analysing the TCGA cohort of patients, we found that PD-L1 and EMT signatures were positively correlated prominently in the ER+ subtype (luminal A and B) but not in ER- ones (Basal or HER2+) (**Figure S5F**). Put together, due to the interconnections among EMT status, prevalence of estrogen signalling and PD-L1, the emergence of reversible drug resistance in ER+ positive breast cancer is likely to lead to higher levels of PD-L1, thus enabling cross-resistance (i.e., tamoxifen-resistant cells being immune evasive) that further promotes their survival.

### Immune Evasive Properties of Hybrid E/M Phenotypes Depends on Transition Trajectories in a Two-Dimensional (2D) EMT Plane

**Figure 5:**
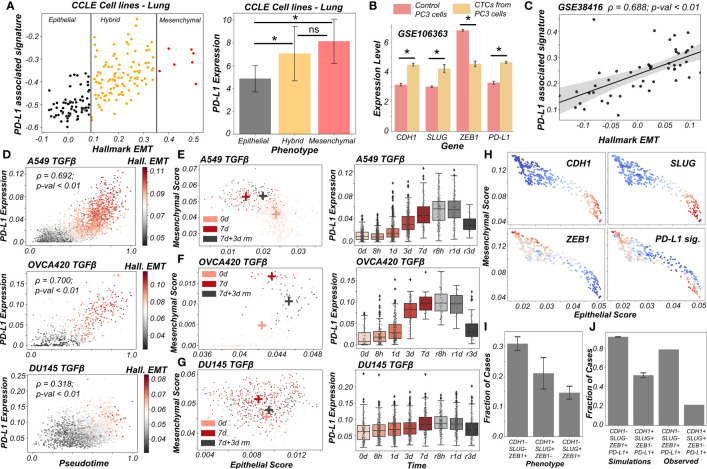
PD-L1 levels depend on the extent and direction of transition of hybrid E/M cells on 2D EM plane **(A)** Scatter plot showing PD-L1 associated and Hallmark EMT signature in lung cancer specific cell lines from CCLE. Boundaries (vertical lines) drawn are based on trisection of the entire range of Hallmark EMT scores of all cell lines in CCLE (left). Quantification of PD-L1 levels of breast cancer cell lines belonging to different EMT status (right). **(B)** Expression levels of ZEB1, SLUG, CDH1 and PD-L1 in control PC3 cell line and PC3 derived CTCs (GSE106363). For **(A, B)**; * denotes a statistically significant difference (p-val < 0.05) between the represented groups assessed by a two-tailed Students t-test assuming unequal variances. **(C)** Scatter plot of PD-L1 associated and Hallmark EMT signature showing a significant positive correlation in single and pooled prostate cancer cells and single DTC from metastatic prostate cancer patients (GSE38416). **(D)** Scatter plot of imputed PD-L1 expression with pseudotime colored by Hallmark EMT scores calculated on imputed gene expression data in TGFβ induced EMT in A549, OVCA420 and DU145 cell lines (GSE147405). **(E–G)** 2D EM plots (left panels) showing cells of 3 different time points (day 0, day 7 and day 3 after TGFβ removal) for 3 different cell lines. + sign indicates the average epithelial and mesenchymal scores of cells belonging to the corresponding time point. Imputed PD-L1 levels over all time points plotted as boxplots (right panels). **(E)** A549 **(F)** OVCA420 **(G)** DU145. **(H)** 2D EM plots of cells from skin squamous cell carcinoma after imputation coloured by CDH1, SLUG, ZEB1 and PD-L1 associated signature. Red represents high expression while blue represents low expression (GSE110357). **(I)** Abundance of top phenotypes in hybrid EM compartment shown in (simulation results). **(J)** Bar plots showing the fraction of different hybrid phenotypes as measured from simulations and seen in experimental data. ns represents results that are not statistically significant (p-val > = 0.05).

To strengthen the association of high PD-L1 levels with hybrid E/M status of cells in other cancers, we probed the CCLE group of cell lines for lung cancer. We found that, as in the case of breast cancer, hybrid E/M cell lines exhibit significantly higher levels of PD-L1 than epithelial ones, but there was not a significant difference between hybrid E/M and mesenchymal cell lines (**Figure 5A**). Furthermore, upon reclassifying CCLE breast cancer cell lines on a two-dimensional epithelial/mesenchymal plane (2D plane) into epithelial, mesenchymal and hybrid EM characteristics, we observed similar trends as were seen when the classification was done by trisecting the range of Hallmark EMT scores (**Figures S6A**, **4E**). This 2D representation allows for deconvoluting and quantifying the activity of epithelial and mesenchymal nature of cells separately, i.e., one can independently monitor a gain of mesenchymal state and loss of epithelial nature during EMT.

Previous experimental and computational efforts has suggested SLUG+CDH1+ cells would display a hybrid E/M phenotype, especially in breast cancer (19, 20). We observed this SLUG+CDH1+ profile in circulating tumour cells (CTCs) obtained from PC3 (a prostate cancer cell line, usually of a more mesenchymal status (48)) (**Figure 5B**; GSE106363). This change in cell phenotype (CTC vs. cell lines) to a more hybrid E/M phenotype is accompanied by a significant increase in PD-L1 levels and concomitant decrease in ZEB1 levels (**Figure 5B**), further strengthening the association of a hybrid EM phenotype with high PD-L1 levels. Further, we analyzed a publicly available expression dataset consisting of single and pooled cell prostate cancer cells along with single disseminated tumour cells. We observed a positive correlation between the Hallmark EMT program and PD-L1 associated gene set (**Figure 5C**) thus supporting our observations at a single-cell level too.

Next, we delved into recent single-cell RNA-seq data that captures hybrid E/M phenotypes (49, 50). We applied imputation algorithms that have been applied to EMT (51) in order to estimate PD-L1 levels in these datasets, and used the imputed levels and/or activity scores for PD-L1 associated genes. We first analyzed the data collected for various cell lines *in vitro* by TGFβ treatment and withdrawal (EMT followed by MET) (49). We observed a strong correlation of PD-L1 levels with pseudo-time. This is, in turn, highly correlated with Hallmark EMT signature, as shown across different cell lines (A549, OVCA420 and DU145) from different cancer types (lung, ovarian and prostate respectively) (**Figure 5D**). Furthermore, the induction of EMT upon treatment was visibly more robust in the case of A549 and OVCA420 in comparison to DU145 cells. This was, in turn, reflected in a weaker correlation of PD-L1 levels with pseudo-time for DU145 cells as compared to other two cases (**Figure 5D**).

To get more robust information about the EMT status of the cells, we plotted cells from control case (day 0), most EMT-like state (day 7 of TGFβ treatment) and subsequent the most MET-like state (3 days after removal of TGFβ post treatment) on the 2D EMT plane (**Figures 5E–G**). On this plane, A549 cells showed both a loss of epithelial traits and a gain of mesenchymal ones as they were treated with TGFβ (**Figure 5E** **;** left). It is important to note here that the 2D plane only captures relative changes in epithelial and mesenchymal nature; without an external reference point, it is difficult to ascertain the absolute EMT status of cells at a given timepoint. Furthermore, upon MET (TGFβ withdrawal), A549 cells partly regained their epithelial nature, without any discernible change in mesenchymal nature. Thus, these cells can be referred to as the hybrid E/M state with respect to the untreated case. Consistently, PD-L1 levels increased as cells progressed through EMT (TGFβ treatment time-points) and decreased as they began their return (TGFβ withdrawal post-treatment). PD-L1 levels in cells 3 days after TGFβ withdrawal (i.e., hybrid E/M) were still higher as compared to untreated cells (**Figure 5E**; right). This suggests that cells fine-tune their PD-L1 levels as they proceed through EMT/MET, thereby enabling hybrid E/M cells to be sufficiently immune-evasive. More interesting trends were seen for the trajectory of OVCA420 cells on the 2D EMT plane (**Figure 5F**) which did not lose any epithelial characteristics upon EMT induction, but gained mesenchymal nature, making the resultant phenotype at day 7 post-treatment a largely hybrid E/M population (**Figure 5F**; left). PD-L1 levels robustly increased, bolstering further evidence for hybrid E/M phenotypes having higher levels of PD-L1 as compared to their more epithelial counterparts (**Figure 5F**; right). As compared to A549 and OVCA420, DU145 cells showcased a much weaker induction of EMT. Consequently, no strong increase in the levels of PD-L1 was observed (**Figure 5G**). This analysis of three different cell lines at multiple time points at an individual-cell level strongly supports our predictions of increased PD-L1 levels being a hallmark of hybrid E/M phenotypes.

Having shown that PD-L1 levels observed in hybrid E/M cells depends on the extent and direction of transitions on a 2D EM plane (**Figure 5G**), we proceeded to interrogate possible mechanistic basis of heterogeneous hybrid E/M phenotypes using data recently reported *in vivo* in squamous cell cancer (50). We applied imputation algorithm MAGIC (51) on the data, we plotted it on 2D EMT plane, and observed an expected negative correlation between the epithelial and mesenchymal programs (**Figures 5H**, **S6B**). A large proportion of cells seen here were CDH1+SLUG+ZEB1- and ZEB1+SLUG-CDH1-, which can be interpreted as two different versions of hybrid E/M phenotypes – the former being relatively more epithelial (“early hybrid”) and the latter being more pushed towards a mesenchymal end (“late hybrid”). Interestingly, CDH1+SLUG-ZEB1- (“strongly epithelial”) and CDH1-SLUG+ZEB1+ (“strongly mesenchymal”) phenotypes were seen only sporadically in this dataset. Among the two hybrid E/M phenotypes, PD-L1 associated gene signature was enriched in a substantial proportion of cells, with a larger number of PD-L1+ cases in the ZEB1+SLUG-CDH1- hybrid subpopulation (**Figure 5H**), thereby identifying context-specific scenarios for heterogeneous hybrid E/M subsets which may manifest immune-evasive properties.

We attempted to offer a mechanistic reason for enrichment of PD-L1+ cases in the ZEB1+SLUG-CDH1-population using our network-based simulations. When we classified all of the steady state solutions by binarizing and considering only the ZEB1, SLUG and CDH1 status in hybrid EM compartment shown in **Figure 1C**, we found that most of the solutions mapped onto a ZEB1+SLUG-CDH1- phenotype closely followed by CDH1+SLUG+ZEB1- phenotype (**Figure 5I**). Furthermore, our parameter-agnostic modelling framework was able to recapitulate the qualitative association of ZEB1+SLUG-CDH1- hybrid E/M phenotype being more likely to be PD-L1+ than the CDH1+ SLUG+ZEB1- hybrid E/M phenotype (**Figure 5J**). While further rigorous experiments are required to substantiate this observation about heterogeneity in hybrid E/M subpopulations in terms of their PD-L1 levels, our analysis elucidates how deciphering the emergent dynamics of the small-scale regulatory networks can explain reported heterogeneity in hybrid E/M state at a single-cell level.

Expression of PD-L1 on tumour cells can cause inhibition of T cell activation/proliferation and result in exhaustion of such T cells (52). To assess whether clinical samples that have intermediate or high EMT scores also have higher expression of T cell exhaustion markers, we calculated the ssGSEA activity scores for a list of 78 gene-signature of T cell exhaustion derived in the context of lung cancer (53) in representative TCGA cancer types shown in **Figure S2**. We found that patient samples with intermediate or high EMT scores consistently showed higher levels of T cell exhaustion markers (**Figure S7**). These observations show promising preliminary evidences that higher PD-L1 expression levels are associated with higher T cell exhaustion markers, and that hybrid E/M tumors can have a large percentage of exhausted T-cells.

## Discussion

Cellular processes are controlled by numerous regulatory feedback loops and mechanisms that maintain a dynamic equilibrium, thus enabling cells to adapt to various internal and external fluctuations. The expression of PD-L1 on cell surface is one such mechanism, that keeps inflammatory responses from uncontrolled activation by providing necessary brakes (2). Tumour cells exploit this check-point to escape from both immunological detection and elimination. PD-L1 on cancer cells’ surface enables them to inhibit T-cell activation, while simultaneously causing them to be exhausted, eventually preventing cancer cells from being targeted by activated T cells (2). High PD-L1 has been exhibited in circulating tumor cells as well across cancers (54, 55), and EMT has been associated with higher PD-L1 levels (5, 56–59). Here, we have investigated PD-L1 levels in hybrid E/M phenotypes, given their higher fitness for metastasis and evasion of various treatment options.

Through *in silico* simulations for underlying networks incorporating crosstalk between PD-L1 and EMT regulators, we observed that hybrid E/M states can show high PD-L1 levels similar to those seen in ‘full EMT’ (mesenchymal) phenotype. This model prediction is substantiated by analysis of gene expression pan-cancer datasets both at individual and bulk RNA-seq levels. We further show that EMT/MET can alter PD-L1 status reversibly in cancer cells, a trend validated in multiple *in vitro* datasets. The hysteretic (non-symmetric) reversibility of EMT dependent acquisition of high PD-L1 levels upon MET could be central to explaining why secondary tumors might have lower PD-L1 levels than primary tumors (14, 15). The extent of loss of PD-L1 upon MET is likely dependent on the magnitude of the MET inducing signal. Intriguingly, while hybrid E/M phenotype was found to be associated with both enhanced PD-L1 and higher stemness, a direct association between PD-L1 levels and stemness was not found. In contrast, residual drug-resistant cells that survive treatment with anti-estrogen therapy in breast cancer are likely to also harbor a hybrid E/M phenotype and higher levels of PD-L1. Our results thus highlight another dimension of the high metastatic fitness of hybrid E/M cells: their immune-suppressive traits.

We acknowledge, as with all models, the limitations of our analysis. We considered here a minimalistic regulatory network that captures key regulatory nodes of interest, and thus is far from being comprehensive. On the one hand, our model recapitulates key observations especially including previously reported associations between EMT and PD-L1 levels; on the other, it provides testable predictions, regarding hybrid E/M states being likely to be PD-L1 positive. The overarching positive correlations between EMT and PD-L1 levels across a majority of the cancers in TCGA shows the broad applicability of our conclusions; also, these were supported in by the analysis of the CCLE and of specific datasets dealing with perturbations. However, our generic model cannot explain some specific exceptions, specifically why certain cancer types such as TGCT, PCPG, SARC and SKCM do not show strong correlation of PD-L1 with either epithelial or mesenchymal programmes. Interestingly, some cancers of mesenchymal origins (LGG, GBM) show positive correlations of PD-L1 with epithelial signatures, suggesting the association of a hybrid E/M state with maximal PD-L1 levels. Finally, our extended model, obtained by considering the additional players OCT4, LIN28, miR-145 and let-7, finds no significant association between stemness and PD-L1 levels. Various additional nodes in the network not considered here may alter this trend, which may then explain previously reported correlations between immune evasion and stemness (60). However, our model predicted an overlap between immune evasion and resistance to targeted therapy in breast cancer. Thus, future efforts are needed to understand these context-specific scenarios in terms of interplay between hybrid E/M phenotypes, stemness, targeted therapy resistance and immune evasion.

**Figure 6:**
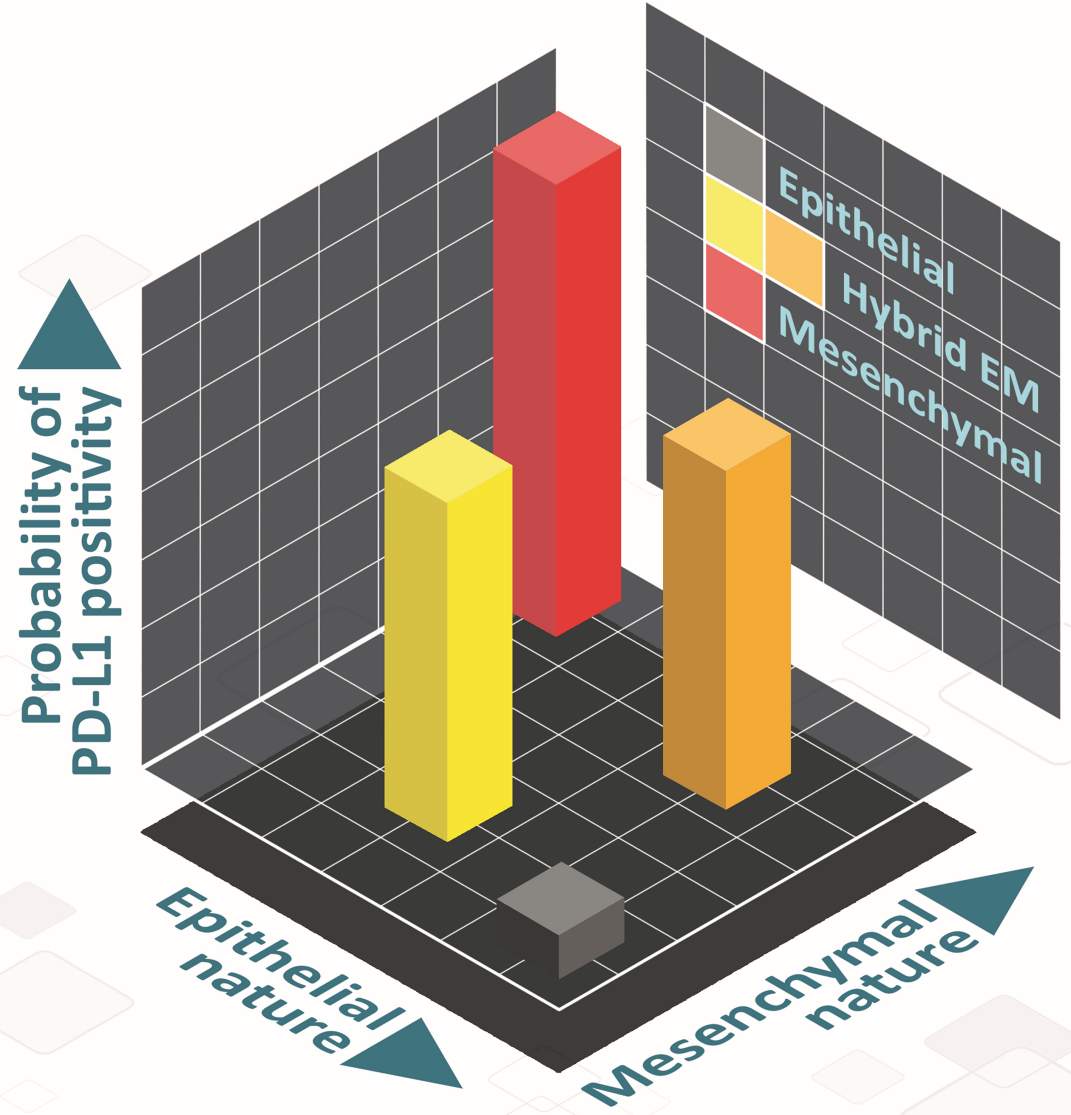
Schematic showing the PD-L1 of hybrid E/M cells on 2D EM plane. Cells can take various paths in this 2D place as they transition from epithelial (grey) to mesenchymal (red), involving different hybrid E/M phenotypes (yellow, orange) – path from grey to yellow to red, and that from grey to orange to red). In hybrid E/M phenotypes, PD-L1 levels are likely to be comparatively higher as compared to that in epithelial ones, and comparable to what is seen for mesenchymal phenotypes.

There has been a recent surge of interest in hybrid E/M phenotypes, and their precise identification is an active area of research, thus calling for more rigorous, preferably quantitative, definition(s). Such definitions, instead of purely descriptive characterization of hybrid E/M cells, can offer new conceptual insights into markers and features of *bona fide* hybrid E/M cells (61). Here, we have used two quantitative strategies based on bulk and single-cell RNA-seq data to demarcate hybrid E/M cells from epithelial and mesenchymal ones. First, we used a two-dimensional metric to quantify EMT (62): epithelial and mesenchymal axes, so that individual changes along those both axes can be deconvoluted and different paths to EMT/MET can be seen, which are beyond the scope for existing scoring metrics for EMT transcriptomes (63). This strategy facilitated us to map changes in PD-L1 levels in cells as a function of their trajectory on the 2D plane and strengthened the association between different possible hybrid E/M state(s) and enhanced PD-L1 levels (**Figure 6**). Second, we defined more absolute boundaries for EMT enrichment using a cohort of CCLE cell lines to classify them into epithelial, hybrid and mesenchymal categories. Such approaches allow us to also characterize underlying manifestations and reasons for heterogeneity within the hybrid E/M phenotypes.

## Materials and Methods

### RACIPE Output of Core Circuit and Its Z-Normalization

RACIPE generates the steady-state values in the log2 scale, which we have further converted into z-scores by using the equation represented in **Eq (1)**. for a better comparative study of the expression of each gene node.


(1) $$Z_{i}=\frac{S_{i}-\bar{S_{m}}}{\sigma_{m}}$$

*Z_i_* = Z normalized expression value,

S_i_ = each steady state value of a given node,

$\bar{S_{m}}$ = combined mean of untransformed expression values.

*σ_m_* = combined standard deviation of untransformed expression values.

We have used all z normalized steady-state solutions up to penta-stable parameter sets for principal component analysis (PCA) plots. PCA was performed on all the z-normalized steady-state solutions. We identified 3 optimal clusters by performing hierarchical clustering on the z- normalized RACIPE data. Contributions of the various node to the principal component axes PC-1 and PC-2 presented in **Table S3**.

For RACIPE, we have used 10,000 parameter sets and 100 initial conditions for each mathematical model to integrate the dynamical equations using Euler’s Method numerically. RACIPE takes a topology file as an input (**Table S1**) and samples the parameters for dynamical simulations from a biologically relevant range (**Table S2**). Depending on the particular parameters, a single model has the potential to give rise to one or more stable steady-state solutions, dependent upon the initial conditions. However, in our current analysis, we have considered up to 5 stable steady-state solutions. As from our initial analysis, we have found a minuscule contribution for >5. We have also performed both overexpression and downexpression of miR-200 and SLUG by 20-fold respectively using RACIPE on the same core circuit.

### EM Score Calculation

The scores were calculated by difference in normalized expression values of node representing mesenchymal (M) and epithelial (E) signatures. EM score = (ZEB1 + SLUG - miR200 - CDH1)/4. Subsequently based on the distribution, cells were categorized into epithelial (<-0.25), hybrid (-0.25 to 0.5), and mesenchymal (>0.5) represented in **Figure 1B**. The same metric was used to calculate EM score for calculation of EM scores for gene regulatory networks shown in **Figures S4C** and **4A**.

### Stemness Circuit and Stemness Score Calculation

We have considered a gene regulatory network shown in **Figure S4C** in which 5 nodes of our core regulatory network are present along with 4 other nodes (OCT4, miR-145, LIN28, and let7) which represents the key players of stemness signature (**Table S1** **)**. The stemness scores (SN) were calculated by difference in normalized expression values of node representing stem-like and non-stem-like signatures: (LIN28 + OCT4 – let7 – miR145)/4. Subsequently based on the distribution, cells were categorized into non-stem-like (SN score <-0.5 and SN score >0.5), and stem-like (SN score = -0.5 to 0.5) represented in **Figure 3C**.

### Stochastic Simulations and Landscape Construction

We simulated the gene regulatory using the Euler-Maruyama method for a representative parameter set (**Table S4**) that showed the co-existence of 3 phenotypes: epithelial with low levels of PD-L1; hybrid E/M with high levels of PD-L1 and mesenchymal with high levels of PD-L1. The corresponding equation is as follows:


$${\text{X}}_{\text{i}}(\text{t}+1)={\text{X}}_{\text{i}}(\text{t})+\Delta \text{t}*{\text{g}}_{{\text{x}}_{\text{i}}}*\prod_{\text{j}}{\text{H}}^{\text{s}}({\text{X}}_{\text{j}}(\text{t}),{\text{X}}_{\text{ji}}0,{\text{n}}_{\text{ji}},\lambda_{\text{ji}})-{\text{k}}_{{\text{xi}}_{\text{i}}}*{\text{X}}_{\text{i}}(\text{t})*\Delta \text{t}+\sqrt{\Delta \text{t}}*\text{N}(0,1)$$

The equation is just a discrete form of the ODE presented before, with an addition of the noise term $\sqrt{\Delta \text{t}}*\text{N}(0,1)$ , where Δt is the time step and N (0,1) is a normal random variable with mean 0 and standard deviation 1. For the parameter set, we simulated the network for 100 different initial conditions sampled uniformly from the range $\left[0,1.5*\frac{{\text{g}}_{{\text{x}}_{\text{i}}}}{{\text{k}}_{{\text{x}}_{\text{i}}}}\right]$ . We then normalized the trajectories using the mean and standard deviation of each node expression obtained from RACIPE and converted the trajectories to EM scores and PD-L1 levels in order to classify them into the observed phenotypes. Using these trajectories, we constructed obtained a probability density (P) of the EM-PD-L1 score pairs and constructed a potential landscape by calculating the pseudo potential as – log (P) (64).

### sRACIPE Simulations

sRACIPE simulations has been performed to generate random set of parameters and to simulate the system with a fixed amount of noise. We have used the webserver of Gene Circuit Explorer (GeneEx) to simulate stochastic time evolution dynamics of our core gene regulatory circuits (34). Parameter values used for the simulation are presented in **Table S5**.

### Gene Expression Data Analysis

Publicly available microarray datasets and RNA-Seq datasets were obtained from GEO. Single-sample gene set enrichment analysis (ssGSEA) (65) was performed on the Hallmark signalling pathways gene signatures from MSigDB (Molecular Signatures Database) (47) to estimate the activity of pathway.

### PD-L1 Associated Signature(s)

To generate PD-L1 associated signatures, we selected top correlated genes (Spearman correlation > 0.5; p-val < 0.01) with PD-L1 levels across at least any 15 out of the 27 cancer types considered for the study (excluding TGCT, PCPG, SARC, SKCM and KIRC as these did not show any consistent association with either the epithelial or mesenchymal metrics). The criteria of 15 out of 27 was relaxed to any 13 out of 27 for analysis of **Figures 5H–J** as the signature from the more stringent signature did not have sufficient power to distinguish the cell populations.

### scRNA Seq Data Analysis

Read counts for scRNA seq datasets were downloaded from GEO datasets and imputation was performed on these datasets using MAGIC algorithm (51). Computation of activity scores of signature gene sets were done using AUCell (66). For computation of epithelial and mesenchymal scores tumour specific gene lists were used from the KS EMT scoring metric (63).

## Data Availability Statement

The original contributions presented in the study are included in the article/**Supplementary Material**. Further inquiries can be directed to the corresponding author. Codes used in manuscript are available at https://github.com/SonaliPNayak/PD-L1_code.

## Author Contributions

SS, SN, KH, PP, AK, and SM performed research. SS and SN prepared first draft of the manuscript; all authors analyzed data and edited the manuscript. MJ and HL designed research. MJ supervised research. All authors contributed to the article and approved the submitted version.

## Funding

MKJ was supported by Ramanujan Fellowship (SB/S2/RJN-049/2018) awarded by Science and Engineering Research Board (SERB), Department of Science & Technology, Government of India. HL was supported by the National Science Foundation sponsored Center for Theoretical Biological Physics – award PHY-2019745, and by PHY-1605817. KH acknowledges support from Prime Ministers’ Research Fellowship (PMRF). SS acknowledges support by KVPY fellowship.

## Conflict of Interest

The authors declare that the research was conducted in the absence of any commercial or financial relationships that could be construed as a potential conflict of interest.

## Publisher’s Note

All claims expressed in this article are solely those of the authors and do not necessarily represent those of their affiliated organizations, or those of the publisher, the editors and the reviewers. Any product that may be evaluated in this article, or claim that may be made by its manufacturer, is not guaranteed or endorsed by the publisher.

## Supplementary Material

Supplementary Figure 1 EMT regulatory network coupled with PD-L1. **(A)** PCA (Principal Component Analysis) plot showing the presence of different clusters emerging from z-normalized scores from RACIPE analysis. Composition of PC1 and PC2 are listed in **Table S3**. **(B)** Hierarchical clustering for z normalized RACIPE output. **(C)** Density histogram of PD-L1 expression fitted with kernel density estimate, showing bimodality. Red lines show the partition between PD-L1 high and PD-L1 low. **(D)** PCA plot coloured by PD-L1 high vs. PD-L1 low levels, showing the enrichment of high PD-L1 levels in hybrid E/M and mesenchymal phenotypes, and that of low PD-L1 levels in an epithelial phenotype. **(E)** Simulation results showing scatter plot of PD-L1 expression with CDH1, ZEB1, and SLUG, as obtained from RACIPE simulations. Spearman’s correlation coefficient (ρ) and corresponding p-value (p-val) are reported. **(F)** Bar graph showing expression of CDH1, SLUG, ZEB1 and PD-L1 in corresponding phenotypes (defined based on EM scores) respectively. **(G)** Scatter plot showing experimental validation from TCGA BRCA – Luminal A cohort of patients of correlations between expression of PD-L1 with CDH1, ZEB1, and SLUG, which was earlier represented in **(E)**. (**H)** Scatter plot showing positive correlation between PD-L1 expression and Hallmark EMT signature in A549 lung adenocarcinoma cells treated with TGFβ to induce EMT over 3 days (GSE17708). * denotes a statistically significant difference (p-val < 0.05) between the represented groups assessed by a two-tailed Students t-test assuming unequal variances.

Supplementary Figure 2 Clinical evidence supporting mathematical model predictions. Scatter plots between expression levels of PD-L1 and Hallmark EMT in representative TCGA cancer types. Spearman’s correlation coefficient (ρ) and corresponding p-value (p-val) are reported.

Supplementary Figure 3 Dynamics upon perturbation of core regulatory network. **(A-C)** Upon miR-200 down expression (DE) and miR-200 over expression (OE): **(A)** density histogram of EM Score fitted with kernel density estimate; **(B)** Scatter plot of PD-L1 expression and EM score; **(C)** Density histogram of PD-L1 expression fitted with kernel density estimate and Bar graph showing change in expression of EM score and PD-L1. **(D-F)** Same as A-C but for SLUG DE and SLUG OE. Horizontal red line shows the partition between PD-L1 expression level being high and low. Vertical red lines show a partition between phenotypes: Epithelial, Hybrid E/M, and Mesenchymal based on EM score. Spearman’s correlation coefficient (ρ) and corresponding p-value (p-val) are given. **(G)** Activity/Expression levels of Hallmark EMT and PD-L1 levels in non-cancerous airway epithelial cells where EMT has been induced (GSE61220). **(H)** Activity/Expression levels of Hallmark EMT and PD-L1 levels in triple negative breast cancer (DKAT) cells grown in either epithelial growth medium (MEGM) or stromal growth medium (SCGM) (GSE33146). **(I)** Expression levels of ZEB1 and PD-L1 in A549 lung cancer cells with EMT induced *via* TGFβ (GSE27473). * denotes a statistically significant difference (p-val < 0.05) between the represented groups assessed by a two-tailed Students t-test assuming unequal variances.

Supplementary Figure 4 Different pathways that may influence the EMT/PD-L1 association. **(A)** Heatmap showing Spearman’s correlation between various signalling pathways and Hallmark EMT/PD-L1 levels respectively. Spearman’s correlation coefficient (ρ) are reported. **(B)** Scatter plots between the Spearman’s correlation of expression levels of PD-L1 and spearman correlation of Hallmark EMT showing the concordance between two heatmaps in **(A)**. **(C)** Schematic representation of stemness circuit diagram with nodes representing various EMT, immune evasion, and stemness signature players. **(D)** Density histogram of Stemness Score (SN score) (LIN28 + OCT4 – let7 – miR145)/4 fitted with kernel density estimate showing predominantly a trimodal distribution. Vertical red lines show the partition between stem-like and non-stem-like based on SN score, where intermediate levels of SN score lie within the ‘stemness window’. **(E)** Scatter plots between expression levels of PD-L1 with iPSC signature and hESC signature respectively in CCLE datasets; signatures are taken from MSigDB.

Supplementary Figure 5 Chacterisation of the association of high PD-L1 levels upon acquisition of a reversible drug resistant phenotype in ER+ breast cancer. **(A)** Heatmap showing stable steady-state solutions for the gene regulatory network shown in Figure4A obtained via RACIPE. **(B)** Frequency density histograms for Resistance score, EM score and PD-L1 levels. The red vertical lines discretise the continuous distributions to distinct phenotypes based on the minima found in the distribution. **(C)** Scatter plot showing a strong association of the EM score with the resistance score followed by classification to 6 possible phenotypes. Spearman’s correlation coefficient (ρ) and corresponding p-value (p-val) are reported. **(D)** Bar plot representing conditional probability of a phenotype being a resistant phenotype given that it belongs to a given EMT status. **(E)** Scatter plot showing correlation between PD-L1 associated gene set and the Hallmark EMT signature in breast cancer specific cell lines from CCLE. The boundaries between epithelial, hybrid and mesenchymal phenotypes are based on trisection of the entire range of Hallmark EMT scores of all cell lines in CCLE. **(F)** Scatter plots between expression levels of PD-L1 and Hallmark EMT across different subtypes in TCGA BRCA cohort of patients. The scatter plot between expression levels of PD-L1 and ESR1 has also been shown for Luminal A subtype of breast cancer. Spearman’s correlation coefficient (ρ) and corresponding p-value (p-val) are reported.

Supplementary Figure 6
**(A)** Classification of CCLE breast cancer cell lines. Scatter plot and quantification of CCLE breast cancer celllines based on the 2D scatter plot of their epithelial and mesenchymal ssGSEA scores. * denotes a statistically significant difference (p-val < 0.05) between the represented groups assessed by a two-tailed Students t-test assuming unequal variances. **(B)** Discretisation of imputed gene expression/activity scores for CDH1, ZEB1, SLUG and PD-L1 associated signature. The cutoff of high vs low is decided based on the minima in the bimodal distributions seen; expect in the case of PD-L1 associated signature, where it is decided as the average of the two relatively shallow minimas in the distribution.

Supplementary Figure 7 T-cell exhaustion in clinical samples correlates with EM status. Violin plots of ssGSEA scores of T-cell exhaustion specific gene list grouped by Hallmark EMT ssGSEA scores in representative TCGA cancer types. Spearman’s correlation coefficient (ρ) and corresponding p-value (p-val) are reported. * denotes a statistically significant difference (p-val < 0.01) between the represented groups assessed by a two-tailed Students t-test assuming unequal variances.
