# Supplementary material for: Immunosuppressive Traits of the Hybrid Epithelial/Mesenchymal Phenotype
Source: Front Immunol. 2021 Dec 15;12:797261. doi: 10.3389/fimmu.2021.797261 (PMC8714906; doi:10.3389/fimmu.2021.797261)
Supplement: Supplementary Figure 1 — EMT regulatory network coupled with PD-L1. (A) PCA (Principal Component Analysis) plot showing the presence of different clusters emerging from z-normalized scores from RACIPE analysis. Composition of PC1 and PC2 are listed in Table S3 . (B) Hierarchical clustering for z normalized RACIPE output. (C) Density histogram of PD-L1 expression fitted with kernel density estimate, showing bimodality. Red lines show the partition between PD-L1 high and PD-L1 low. (D) PCA plot coloured by PD-L1 high vs. PD-L1 low levels, showing the enrichment of high PD-L1 levels in hybrid E/M and mesenchymal phenotypes, and that of low PD-L1 levels in an epithelial phenotype. (E) Simulation results showing scatter plot of PD-L1 expression with CDH1, ZEB1, and SLUG, as obtained from RACIPE simulations. Spearman’s correlation coefficient (ρ) and corresponding p-value (p-val) are reported. (F) Bar graph showing expression of CDH1, SLUG, ZEB1 and PD-L1 in corresponding phenotypes (defined based on EM scores) respectively. (G) Scatter plot showing experimental validation from TCGA BRCA – Luminal A cohort of patients of correlations between expression of PD-L1 with CDH1, ZEB1, and SLUG, which was earlier represented in (E). (H) Scatter plot showing positive correlation between PD-L1 expression and Hallmark EMT signature in A549 lung adenocarcinoma cells treated with TGFβ to induce EMT over 3 days (GSE17708). * denotes a statistically significant difference (p-val < 0.05) between the represented groups assessed by a two-tailed Students t-test assuming unequal variances. [file Presentation_1.pdf]

Supplemental Information

Text S1. ODE model

The chemical rate equation given below is a generic representation of the rate of change of the expression or level of each element (node).

$$\dot{E} = g_E * H^s(B, B_0, n, \lambda_{B,E}) - k_E * E \quad \text{(S1)}$$

Where g_E is the basal production rate of E and k_E is the innate degradation rate of E, where E represents expression level of a particular element (node). H^S is the shifted Hill function, representing each interaction or regulatory term in the gene regulatory network between a pair of nodes, where H^S represents interaction of node B affecting the production of node E is defined as follows:

$$H^s(B, \lambda) = H^-(B) + \lambda H^+(B) \quad \text{(S2)}$$

$$H^-(B) = \frac{1}{(1 + \left(\frac{B}{B_0}\right)^{nB})}$$

$$H^+(B) = 1 - H^-(B)$$

B_o = threshold value for that interaction,

n = cooperativity for that interaction,

λ = fold change from the basal synthesis rate of E due to B.

Hence, λ > 1 for activators and λ < 1 for inhibitors.

$$\frac{d\mathrm{ZEB1}}{dt} = \mathrm{g}_{\mathrm{ZEB1}} * \mathrm{H}^s(\mathrm{miR200}, \lambda_{\mathrm{miR200,ZEB1}}) * \mathrm{H}^s(\mathrm{ZEB1}, \lambda_{\mathrm{ZEB1,ZEB1}}) * \mathrm{H}^s(\mathrm{CDH1}, \lambda_{\mathrm{CDH1,ZEB1}}) * \mathrm{H}^s(\mathrm{SLUG}, \lambda_{\mathrm{SLUG,ZEB1}}) - k_{\mathrm{ZEB1}} * \mathrm{ZEB1} \quad \text{(S3)}$$

$$\frac{d\mathrm{miR200}}{dt} = \mathrm{g}_{\mathrm{miR200}} * \mathrm{H}^s(\mathrm{ZEB1}, \lambda_{\mathrm{ZEB1,miR200}}) * \mathrm{H}^s(\mathrm{SLUG}, \lambda_{\mathrm{SLUG,miR200}}) - k_{\mathrm{miR200}} * \mathrm{miR200} \quad \text{(S4)}$$

$$\frac{d\mathrm{PDL1}}{dt} = \mathrm{g}_{\mathrm{PDL1}} * \mathrm{H}^s(\mathrm{PDL1}, \lambda_{\mathrm{miR200,PDL1}}) - k_{\mathrm{PDL1}} * \mathrm{PDL1} \quad \text{(S5)}$$

$$\frac{d\mathrm{CDH1}}{dt} = \mathrm{g}_{\mathrm{CDH1}} * \mathrm{H}^s(\mathrm{PDL1}, \lambda_{\mathrm{PDL1,CDH1}}) * \mathrm{H}^s(\mathrm{ZEB1}, \lambda_{\mathrm{ZEB1,CDH1}}) * \mathrm{H}^s(\mathrm{SLUG}, \lambda_{\mathrm{SLUG,CDH1}}) - k_{\mathrm{CDH1}} * \mathrm{CDH1} \quad \text{(S6)}$$

$$\frac{d\mathrm{SLUG}}{dt} = \mathrm{g}_{\mathrm{SLUG}} * \mathrm{H}^s(\mathrm{miR200}, \lambda_{\mathrm{miR200,SLUG}}) * \mathrm{H}^s(\mathrm{SLUG}, \lambda_{\mathrm{SLUG,SLUG}}) - k_{\mathrm{SLUG}} * \mathrm{SLUG} \quad \text{(S7)}$$

S. No	Interactions	Reference
**1**	ZEB1, miR200 mutual inhibition	(1)
**2**	ZEB1 self-activation	(2)
**3**	miR200 downregulate PDL1	(3)
**4**	PD-L1 downregulate CDH1	(4)
**5**	ZEB1, CDH1 mutual inhibition	(5–7)
**6**	SLUG, miR200 mutual inhibition	(8)
**7**	SLUG upregulate ZEB1	(9)
**8**	SLUG downregulate CDH1	(10)
**9**	SLUG self-activation	(11)
**10**	OCT4 self-activation	(12)
**11**	OCT4 upregulates miR-200	(13)
**12**	OCT4 upregulates SLUG	(14)
**13**	PD-L1 upregulate OCT4	(15)
**14**	LIN28 upregulate OCT4	(16)
**15**	OCT4, miR-145 mutual inhibition	(13)
**16**	let7 self-activation	(16)
**17**	let7 downregulate PD-L1	(17)
**18**	let7 downregulate ZEB1	(16)
**19**	let7, LIN28 mutual inhibition	(16)
**20**	LIN28 self-activation	(16)
**21**	miR-200 downregulates LIN28	(16)
**22**	miR-145, ZEB1 mutual inhibition	(18)
**23**	miR-145, SLUG mutual inhibition	(19, 20)
**24**	SLUG, ERα66 mutual inhibition	(21)
**25**	ZEB1 downregulate ERα66	(21)
**26**	ERα66 self-activation	(21)
**27**	ERα66 downregulate ERα36	(21)
**28**	ERα66 downregulate PD-L1	(22)
**29**	ERα36 upregulate ZEB1	(21)

Table S1*: Interactions for core GRN (S. No 1-11), Interactions for stemness circuit (S. No 1-23), Interactions for drug resistance circuit (S. No 1-11 & S. No 24-29).*

Parameters	Minimum - Maximum (Uniform Values)
**Maximum production rate (*g*)**	1-100
**Degradation rate (*k*)**	0.1-1
**Fold change (*λ*)**	1-100
**Threshold (B_o)**	The ranges depend on the inward regulations, which are estimated by a Monte Carlo simulation.
**Hill coefficient (*n*)**	1-6

***Table S2**: Ranges of the parameters within which RACIPE randomly samples.*

Wcoeff	ZeB1	miR200	SLUG	CDH1	PDL1
**PC1(84.44%)**	0.46487	-0.146355	-0.34263	-0.10234	0.79661
**PC2(7.36%)**	-0.45943	0.21006	0.12549	0.72337	0.45361

***Table S3**: Contributions of the various node to the principal component axes PC-1 and PC-2.*

Parameter	S. No. 1	Parameter	S. No. 1
Prod_of_ZEB1	51.20536	Inh_of_SLUGTomiR200	0.01051
Prod_of_miR200	73.74496	Trd_of_miR200ToPDL1	9.788561
Prod_of_PDL1	43.96804	Num_of_miR200ToPDL1	6
Prod_of_CDH1	89.20117	Inh_of_miR200ToPDL1	0.01356
Prod_of_SLUG	29.23346	Trd_of_PDL1ToCDH1	34.06882
Deg_of_ZEB1	0.62873	Num_of_PDL1ToCDH1	2
Deg_of_miR200	0.861129	Inh_of_PDL1ToCDH1	0.010248
Deg_of_PDL1	0.837638	Trd_of_ZEB1ToCDH1	0.80368
Deg_of_CDH1	0.192489	Num_of_ZEB1ToCDH1	3
Deg_of_SLUG	0.549676	Inh_of_ZEB1ToCDH1	0.041006
Trd_of_miR200ToZEB1	6.155112	Trd_of_SLUGToCDH1	11.37985
Num_of_miR200ToZEB1	2	Num_of_SLUGToCDH1	6
Inh_of_miR200ToZEB1	0.014161	Inh_of_SLUGToCDH1	0.06103
Trd_of_ZEB1ToZEB1	0.662827	Trd_of_miR200ToSLUG	8.200188
Num_of_ZEB1ToZEB1	2	Num_of_miR200ToSLUG	3
Act_of_ZEB1ToZEB1	54.05437	Inh_of_miR200ToSLUG	0.16813
Trd_of_CDH1ToZEB1	0.547427	Trd_of_SLUGToSLUG	13.8752
Num_of_CDH1ToZEB1	2	Num_of_SLUGToSLUG	3
Inh_of_CDH1ToZEB1	0.01252	Act_of_SLUGToSLUG	25.64712
Trd_of_SLUGToZEB1	10.8639	Num_of_ZEB1TomiR200	1
Num_of_SLUGToZEB1	5	Inh_of_ZEB1TomiR200	0.018446
Act_of_SLUGToZEB1	72.00952	Trd_of_SLUGTomiR200	5.575322
Trd_of_ZEB1TomiR200	1.573366	Num_of_SLUGTomiR200	6

Table S4*: Parameter values for generation of free energy landscape and steady state plot (Fig. 2A, B). Here, production terms are represented in green color, degradation terms are represented in orange color, Hill coefficients represented in cyan color, Threshold terms represented in pink color and fold change represented in white color.*

Parameters	Values	Parameters	Values
G_ZEB1	62.16564	FC_ZEB1_ZEB1	68.19913
G_miR200	74.63792	FC_miR200_ZEB1	91.63278
G_PDL1	44.80946	FC_CDH1_ZEB1	76.93204
G_CDH1	80.99668	FC_SLUG_ZEB1	97.67022
G_SLUG	32.3487	FC_ZEB1_miR200	67.84465
K_ZEB1	0.529706	FC_SLUG_miR200	67.49304
K_miR200	0.330501	FC_miR200_PDL1	68.41486
K_PDL1	0.928506	FC_ZEB1_CDH1	94.1392
K_CDH1	0.88837	FC_PDL1_CDH1	38.9354
K_SLUG	0.518909	FC_SLUG_CDH1	73.84352
TH_ZEB1_ZEB1	0.902465	FC_miR200_SLUG	56.15421
TH_miR200_ZEB1	4.090531	FC_SLUG_SLUG	72.962
TH_CDH1_ZEB1	0.623593	N_miR200_ZEB1	4
TH_SLUG_ZEB1	2.210129	N_CDH1_ZEB1	3
TH_ZEB1_miR200	0.814146	N_SLUG_ZEB1	2
TH_SLUG_miR200	6.22973	N_ZEB1_miR200	5
TH_miR200_PDL1	3.811111	N_SLUG_miR200	4
TH_ZEB1_CDH1	0.762967	N_miR200_PDL1	2
TH_PDL1_CDH1	9.033578	N_ZEB1_CDH1	1
TH_SLUG_CDH1	12.66515	N_PDL1_CDH1	3
TH_miR200_SLUG	8.263242	N_SLUG_CDH1	1
TH_SLUG_SLUG	8.61641	N_miR200_SLUG	5
N_ZEB1_ZEB1	2	N_SLUG_SLUG	1

***Table S5**: Basic parameter values. The parameters were adopted from sRACIPE (**Fig. 2C**), which generate random set of parameters and to simulate the system with a fixed amount of noise. In the table production terms are represented in green color, degradation terms are represented in orange color, Hill coefficients represented in cyan color, Threshold terms represented in pink color and fold change represented in white color.*

Tissue	Number of cell lines	Spearman's correlation	log10(P-value)
**PROSTATE**	8	0.714285714	1.332283447
**LIVER**	28	0.695675972	4.402583271
**BILIARYTRACT**	8	0.69047619	1.236644508
**SOFTTISSUE**	21	0.677922078	3.135173547
**BREAST**	59	0.654997078	7.739785255
**PLEURA**	11	0.636363636	1.452385494
**KIDNEY**	36	0.628828829	4.397764365
**AUTONOMICGANGLIA**	17	0.593137255	1.917750607
**LUNG**	187	0.515031619	13.33278092
**LARGEINTESTINE**	61	0.495769434	4.314209954
**BONE**	29	0.44137931	1.781603994
**OVARY**	52	0.433791514	2.881139725
**ENDOMETRIUM**	27	0.415140415	1.504534345
**URINARYTRACT**	27	0.368131868	1.230258399
**HAEMATOPOIETICANDLYMPHOIDTISE**	180	0.342856261	5.609133823
**STOMACH**	38	0.333406281	1.389322095
**PANCREAS**	44	0.286257928	1.224801918
**CENTRALNERVOUSSYSTEM**	69	0.242053343	1.34592534
**SKIN**	62	0.143763693	0.57683202
**OESOPHAGUS**	26	0.140512821	0.306655414
**THYROID**	12	0.048951049	0.055557512
**UPPERAERODIGESTIVETRACT**	32	0.02016129	0.039630176

***Table S6**: Tissue specific CCLE spearman’s correlation and p values.*

References

1. U. Burk, *et al.*, A reciprocal repression between ZEB1 and members of the miR-200 family promotes EMT and invasion in cancer cells. *EMBO reports* **9**, 582–589 (2008).

2. L. Hill, G. Browne, E. Tulchinsky, ZEB/miR-200 feedback loop: At the crossroads of signal transduction in cancer. *International Journal of Cancer* **132**, 745–754 (2013).

3. L. Chen, *et al.*, Metastasis is regulated via microRNA-200/ZEB1 axis control of tumour cell PD-L1 expression and intratumoral immunosuppression. *Nature Communications* **5**, 5241 (2014).

4. W. Yu, *et al.*, PD-L1 promotes tumor growth and progression by activating WIP and β-catenin signaling pathways and predicts poor prognosis in lung cancer. *Cell Death & Disease* **11**, 506 (2020).

5. A. B. Singh, *et al.*, Claudin-1 Up-regulates the Repressor ZEB-1 to Inhibit E-Cadherin Expression in Colon Cancer Cells. *Gastroenterology* **141**, 2140–2153 (2011).

6. Y.-F. Tan, *et al.*, β-catenin-coordinated lncRNA MALAT1 up-regulation of ZEB-1 could enhance the telomerase activity in HGF-mediated differentiation of bone marrow mesenchymal stem cells into hepatocytes. *Pathology - Research and Practice* **215**, 546–554 (2019).

7. S. Orsulic, O. Huber, H. Aberle, S. Arnold, R. Kemler, E-cadherin binding prevents beta-catenin nuclear localization and beta-catenin/LEF-1-mediated transactivation. *Journal of Cell Science* **112**, 1237–1245 (1999).

8. Y.-N. Liu, *et al.*, MiR-1 and miR-200 inhibit EMT via Slug-dependent and tumorigenesis via Slug-independent mechanisms. *Oncogene* **32**, 296–306 (2013).

9. C. Wels, S. Joshi, P. Koefinger, H. Bergler, H. Schaider, Transcriptional activation of ZEB1 by Slug leads to cooperative regulation of the epithelial-mesenchymal transition-like phenotype in melanoma. *The Journal of investigative dermatology* **131**, 1877–1885 (2011).

10. K. M. Hajra, D. Y.-S. Chen, E. R. Fearon, The SLUG zinc-finger protein represses E-cadherin in breast cancer. *Cancer research* **62**, 1613–1618 (2002).

11. B. Kumar, *et al.*, Auto-regulation of Slug mediates its activity during epithelial to mesenchymal transition. *Biochimica et biophysica acta* **1849**, 1209–1218 (2015).

12. G. Shi, Y. Jin, Role of Oct4 in maintaining and regaining stem cell pluripotency. *Stem Cell Research & Therapy* **1**, 39 (2010).

13. M. K. Jolly, *et al.*, Stability of the hybrid epithelial/mesenchymal phenotype. *Oncotarget; Vol 7, No 19* (2016).

14. G. Mandal, *et al.*, Heterodimer formation by Oct4 and Smad3 differentially regulates epithelial-to-mesenchymal transition-associated factors in breast cancer progression. *Biochimica et Biophysica Acta (BBA) - Molecular Basis of Disease* **1864**, 2053–2066 (2018).

15. S. Almozyan, *et al.*, PD-L1 promotes OCT4 and Nanog expression in breast cancer stem cells by sustaining PI3K/AKT pathway activation. *International journal of cancer* **141**, 1402–1412 (2017).

16. M. K. Jolly, *et al.*, Coupling the modules of EMT and stemness: A tunable "stemness window" model. *Oncotarget* **6**, 25161–25174 (2015).

17. Y. Chen, *et al.*, LIN28/let-7/PD-L1 Pathway as a Target for Cancer Immunotherapy. *Cancer Immunology Research* **7**, 487 LP – 497 (2019).

18. K. Hari, *et al.*, Identifying inhibitors of epithelial–mesenchymal plasticity using a network topology-based approach. *npj Systems Biology and Applications* **6**, 15 (2020).

19. L.-L. Mei, *et al.*, miR-145-5p Suppresses Tumor Cell Migration, Invasion and Epithelial to Mesenchymal Transition by Regulating the Sp1/NF-κB Signaling Pathway in Esophageal Squamous Cell Carcinoma. *International journal of molecular sciences* **18**, 1833 (2017).

20. V. J. Findlay, *et al.*, SNAI2 modulates colorectal cancer 5-fluorouracil sensitivity through miR145 repression. *Molecular cancer therapeutics* **13**, 2713–2726 (2014).

21. S. Sahoo, *et al.*, A mechanistic model captures the emergence and implications of non-genetic heterogeneity and reversible drug resistance in ER+ breast cancer cells. *NAR Cancer* **3** (2021).

22. L. Liu, *et al.*, ERα is a negative regulator of PD-L1 gene transcription in breast cancer. *Biochemical and biophysical research communications* **505**, 157–161 (2018).

Immunosuppressive Traits of the Hybrid Epithelial/Mesenchymal Phenotype

Supplementary figures

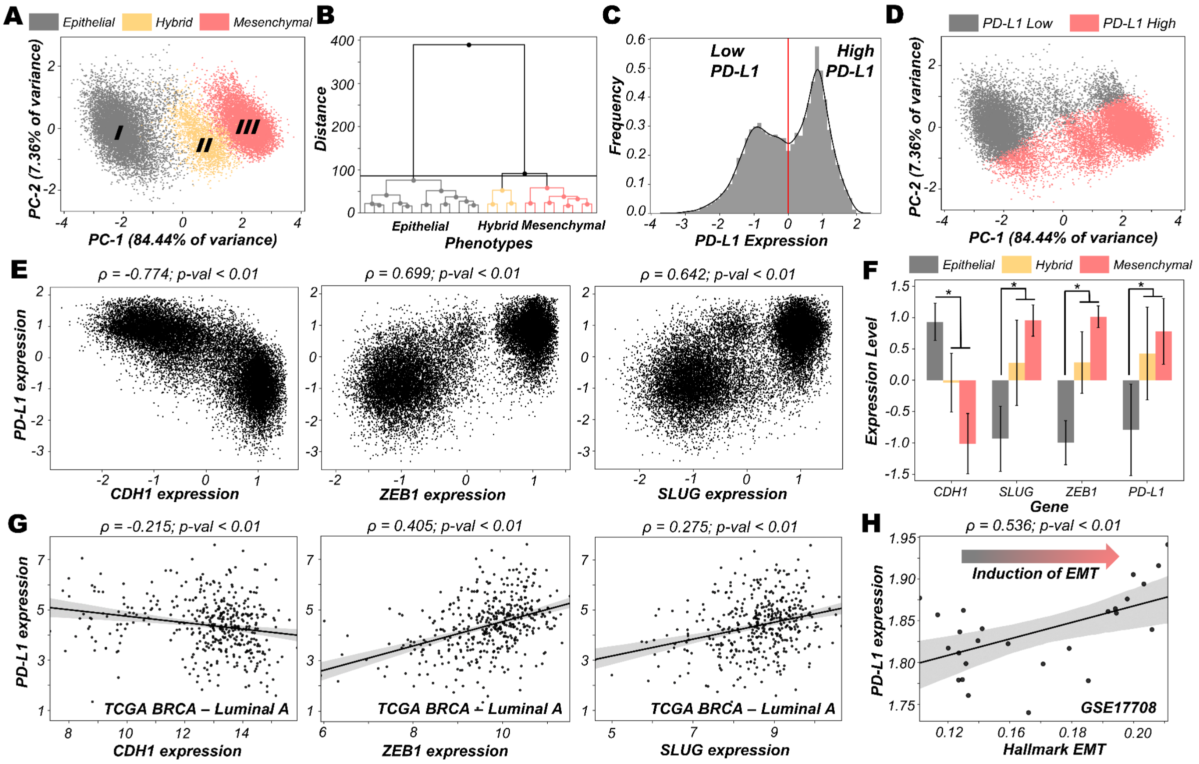

Fig S1. EMT regulatory network coupled with PD-L1. A) *PCA (Principal Component Analysis) plot showing the presence of different clusters emerging from z-normalised scores from RACIPE analysis. Composition of PC1 and PC2 are listed in Table S3.* ***B)*** *Hierarchical clustering for z normalised RACIPE output.* ***C)*** *Density histogram of PD-L1 expression fitted with kernel density estimate, showing bimodality. Red lines show the partition between PD-L1 high and PD-L1 low.* ***D)*** *PCA plot coloured by PD-L1 high vs. PD-L1 low levels, showing the enrichment of high PD-L1 levels in hybrid E/M and mesenchymal phenotypes, and that of low PD-L1 levels in an epithelial phenotype.* ***E)*** *Simulation results showing scatter plot of PD-L1 expression with CDH1, ZEB1, and SLUG, as obtained from RACIPE simulations. Spearman's correlation coefficient (ρ) and corresponding p-value (p-val) are reported.* ***F)*** *Bar graph showing expression of CDH1, SLUG, ZEB1 and PD-L1 in corresponding phenotypes (defined based on EM scores) respectively.* ***G)*** *Scatter plot showing experimental validation from TCGA BRCA – Luminal A cohort of patients of correlations between expression of PD-L1 with CDH1, ZEB1, and SLUG, which was earlier represented in E).* ***H)*** *Scatter plot showing positive correlation between PD-L1 expression and Hallmark EMT signature in A549 lung adenocarcinoma cells treated with TGFβ to induce EMT over 3 days (GSE17708). * denotes a statistically significant difference (p-val < 0.05) between the represented groups assessed by a two-tailed Students t-test assuming unequal variances.*

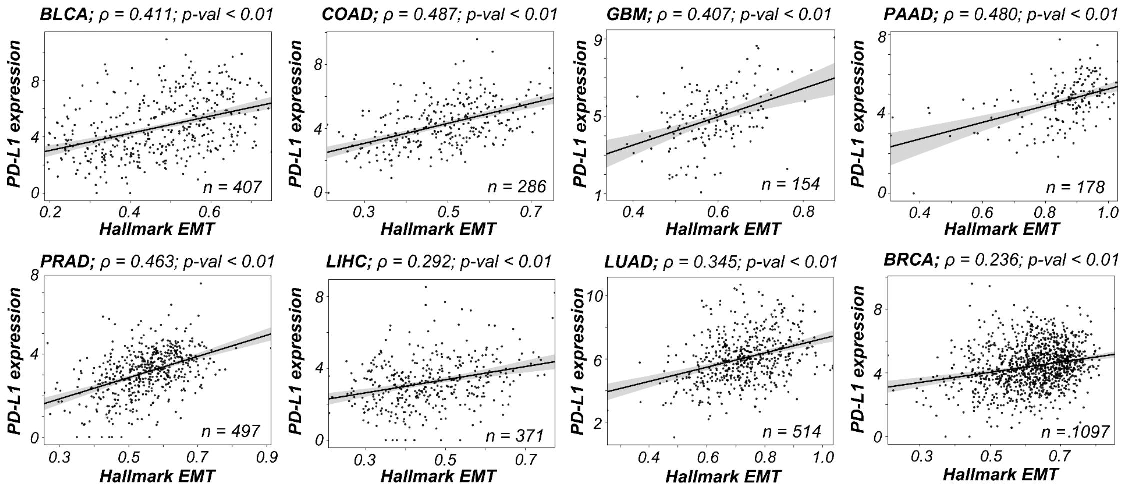

Fig S2. Clinical evidence supporting mathematical model predictions. *Scatter plots between expression levels of PD-L1 and Hallmark EMT in representative TCGA cancer types. Spearman's correlation coefficient (ρ) and corresponding p-value (p-val) are reported.*

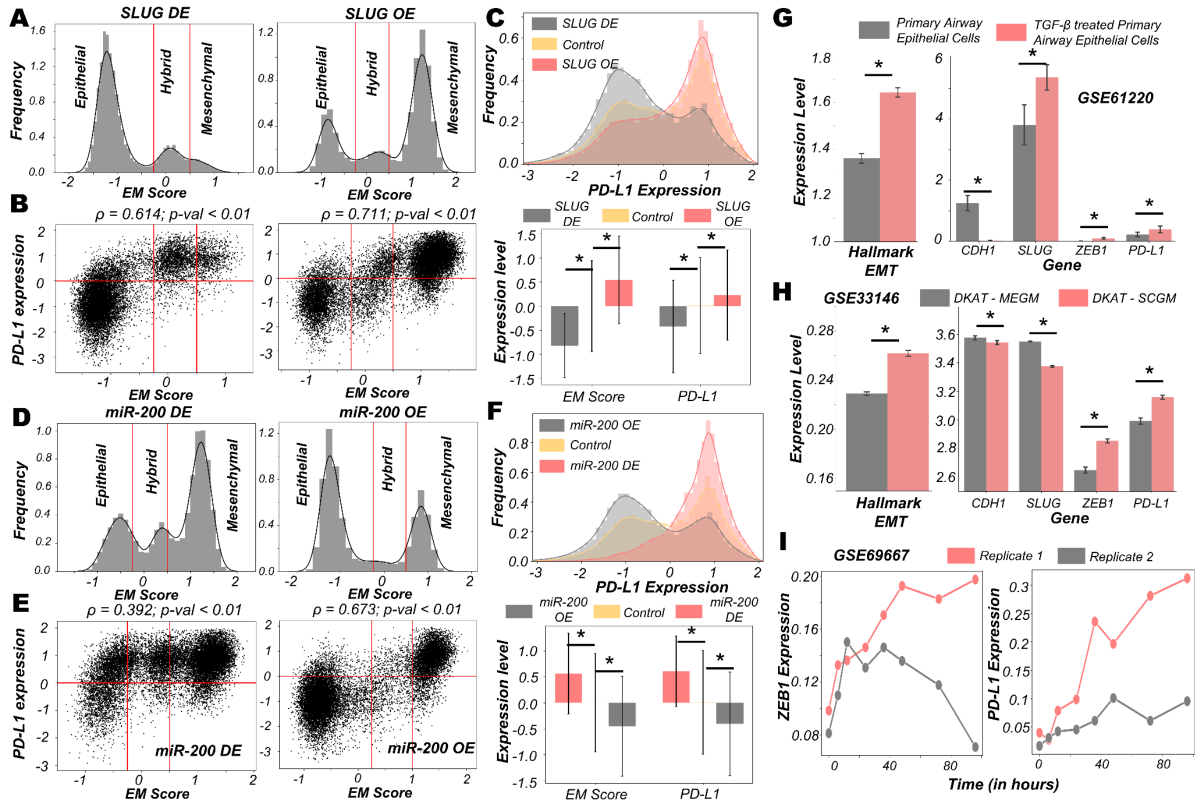

Fig S3. Dynamics upon perturbation of core regulatory network. A-C) *Upon miR-200 down expression (DE) and miR-200 over expression (OE):* ***A)*** *density histogram of EM Score fitted with kernel density estimate;* ***B)*** *Scatter plot of PD-L1 expression and EM score;* ***C)*** *Density histogram of PD-L1 expression fitted with kernel density estimate and Bar graph showing change in expression of EM score and PD-L1.* ***D-F)*** *Same as A-C but for SLUG DE and SLUG OE. Horizontal red line shows the partition between PD-L1 expression level being high and low. Vertical red lines show a partition between phenotypes: Epithelial, Hybrid E/M, and Mesenchymal based on EM score. Spearman's correlation coefficient (ρ) and corresponding p-value (p-val) are given.* ***G)*** *Activity/Expression levels of Hallmark EMT and PD-L1 levels in non-cancerous airway epithelial*

cells where EMT has been induced (GSE61220). ***H)*** *Activity/Expression levels of Hallmark EMT and PD-L1 levels in triple negative breast cancer (DKAT) cells grown in either epithelial growth medium (MEGM) or stromal growth medium (SCGM) (GSE33146).* ***I)*** *Expression levels of ZEB1 and PD-L1 in A549 lung cancer cells with EMT induced via TGFβ (GSE27473). * denotes a statistically significant difference* (p-val < 0.05) *between* the represented groups *assessed by a two-tailed Students t-test assuming unequal variances.*

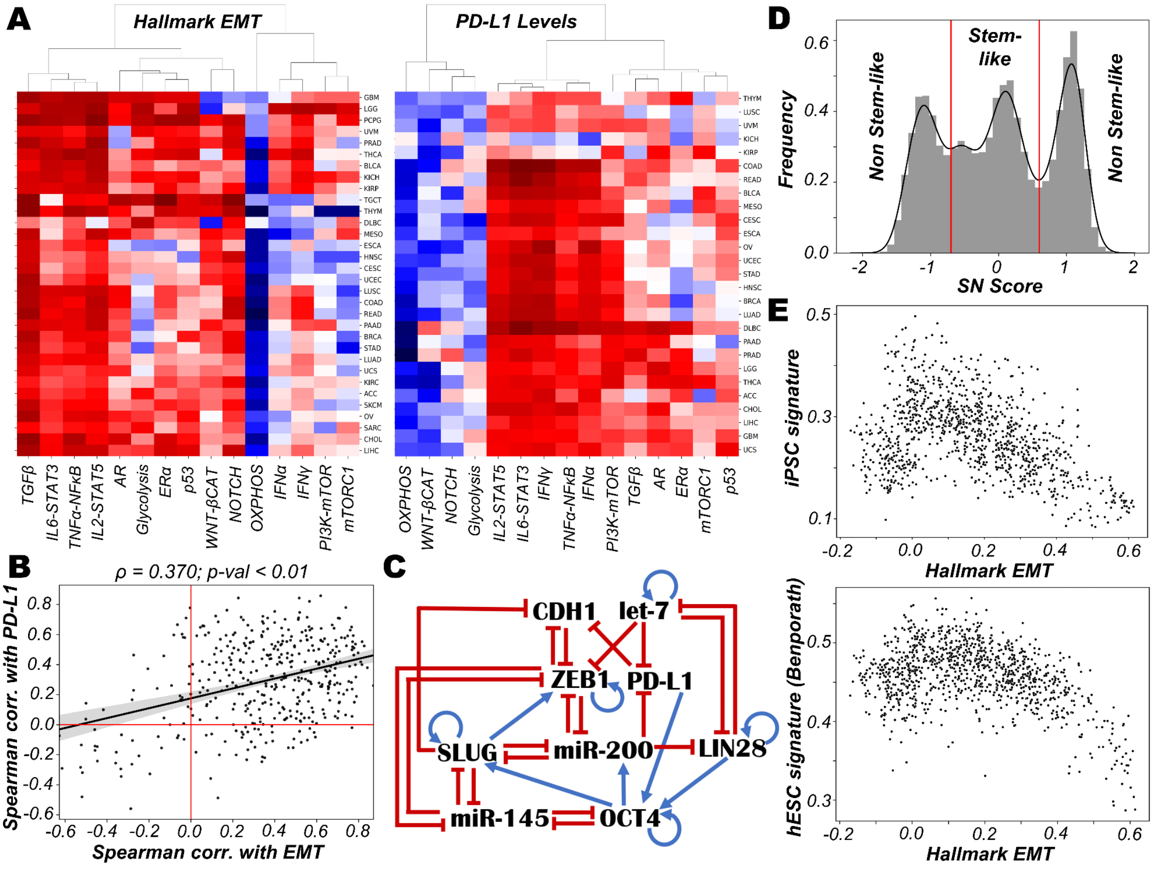

Fig S4. Different pathways that may influence the EMT/PD-L1 association. A) *Heatmap showing Spearman's correlation between various signalling pathways and Hallmark EMT/PD-L1 levels respectively. Spearman's correlation coefficient (ρ) and e corresponding p-value (p-val) are reported.* ***B)*** *Scatter plots between the Spearman's correlation of expression levels of PD-L1 and spearman correlation of Hallmark EMT showing the concordance between two heatmaps in (A).* ***C)*** *Schematic representation of stemness circuit diagram with nodes representing various EMT, immune evasion, and stemness signature players.* ***(D)*** *Density histogram of Stemness Score (SN score) (LIN28 + OCT4 – let7 – miR145)/4 fitted with kernel density estimate showing predominantly a trimodal distribution. Vertical red lines show the partition between stem-like and non-stem-like based on SN score, where intermediate levels of SN score lie within the 'stemness window'.* ***E)*** *Scatter plots between expression levels of PD-L1 with iPSC signature and hESC signature (Ben-porath et al. 2008) respectively in CCLE datasets.*

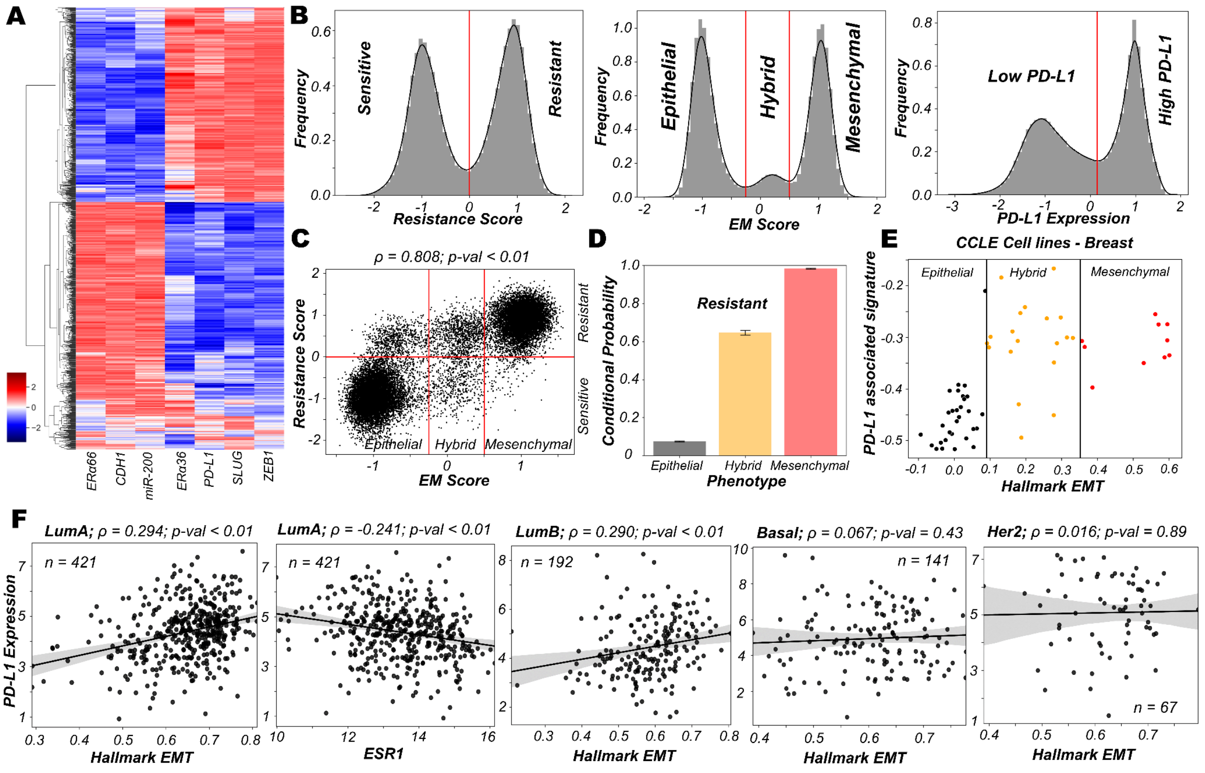

Fig S5. Chacterisation of the association of high PD-L1 levels upon acquisition of a reversible drug resistant phenotype in ER+ breast cancer. A) *Heatmap showing stable steady-state solutions for the gene regulatory network shown in Fig 4A obtained via RACIPE.* ***B)*** *Frequency density histograms for Resistance score, EM score and PD-L1 levels. The red vertical lines discretise the continuous distributions to distinct phenotypes based on the minima found in the distribution.* ***C)*** *Scatter plot showing a strong association of the EM score with the resistance score followed by classification to 6 possible phenotypes. Spearman's correlation coefficient (ρ) and corresponding p-value (p-val) are reported.* ***D)*** *Bar plot representing conditional probability of a phenotype being a resistant phenotype given that it belongs to a given EMT status.* ***E)*** *Scatter plot showing correlation between PD-L1 associated gene set and the Hallmark EMT signature in breast cancer specific cell lines from CCLE. The boundaries between epithelial, hybrid and mesenchymal phenotypes are based on trisection of the entire range of Hallmark EMT scores of all cell lines in CCLE.* ***F)*** *Scatter plots between expression levels of PD-L1 and Hallmark EMT across different subtypes in TCGA BRCA cohort of patients. The scatter plot between expression levels of PD-L1 and ESR1 has also been shown for Luminal A subtype of breast cancer. Spearman's correlation coefficient (ρ) and corresponding p-value (p-val) are reported.*

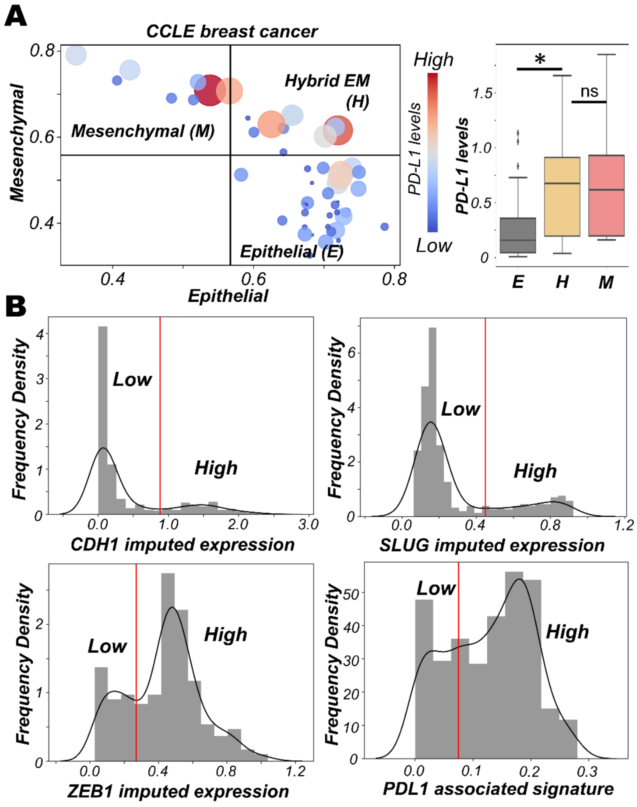

Fig S6. A) *Classification of CCLE breast cancer cell lines. Scatter plot and quantification of CCLE breast cancer celllines based on the 2D scatter plot of their epithelial and mesenchymal ssGSEA scores. * denotes a statistically significant difference (p-val < 0.05) between the represented groups assessed by a two-tailed Students t-test assuming unequal variances.* ***B)*** *Discretisation of imputed gene expression/activity scores for CDH1, ZEB1, SLUG and PD-L1 associated signature. The cutoff of high vs low is decided based on the minima in the bimodal distributions seen; expect in the case of PD-L1 associated signature, where it is decided as the average of the two relatively shallow minimas in the distribution.*

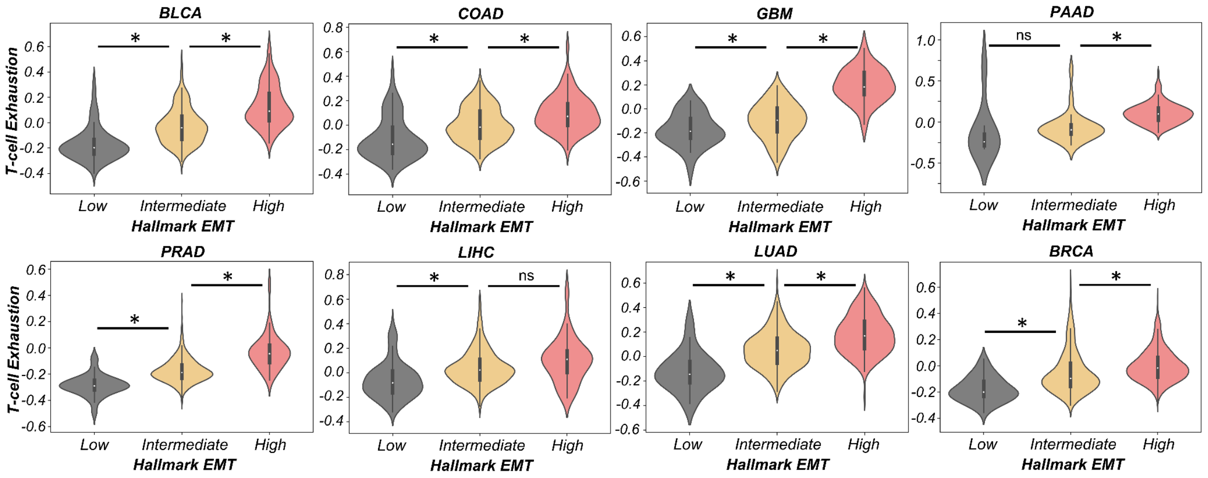

Fig S7. T-cell exhaustion in clinical samples correlates with EM status. *Violin plots of ssGSEA scores of T-cell exhaustion specific gene list grouped by Hallmark EMT ssGSEA scores in representative TCGA cancer types. Spearman's correlation coefficient (ρ) and corresponding p-value (p-val) are reported. * denotes a statistically significant difference (p-val < 0.01) between the represented groups assessed by a two-tailed Students t-test assuming unequal variances.*
